# ANGPTL2 increases bone metastasis of breast cancer cells through enhancing CXCR4 signaling

**DOI:** 10.1038/srep09170

**Published:** 2015-03-16

**Authors:** Tetsuro Masuda, Motoyoshi Endo, Yutaka Yamamoto, Haruki Odagiri, Tsuyoshi Kadomatsu, Takayuki Nakamura, Hironori Tanoue, Hitoshi Ito, Masaki Yugami, Keishi Miyata, Jun Morinaga, Haruki Horiguchi, Ikuyo Motokawa, Kazutoyo Terada, Masaki Suimye Morioka, Ichiro Manabe, Hirotaka Iwase, Hiroshi Mizuta, Yuichi Oike

**Affiliations:** 1 Department of Molecular Genetics, Graduate School of Medical Sciences, Kumamoto University, 1-1-1 Honjo, Chuo-ku, Kumamoto 860-8556, Japan; 2 Department of Orthopaedic Surgery, Graduate School of Medical Sciences, Kumamoto University, 1-1-1 Honjo, Chuo-ku, Kumamoto 860-8556, Japan; 3 Department of Molecular-Targeting Therapy for Breast Cancer, Kumamoto University Hospital, Kumamoto University, 1-1-1 Honjo, Chuo-ku, Kumamoto 860-8556, Japan; 4 Institute of Resource Development and Analysis, Kumamoto University, 1-1-1 Honjo, Chuo-ku, Kumamoto 860-8556, Japan; 5 Department of Cardiovascular Medicine, Graduate School of Medicine, The University of Tokyo, 7-3-1 Hongo, Bunkyo-ku, Tokyo 113-8655, Japan; 6 Department of Breast and Endocrine Surgery, Graduate School of Medical Sciences, Kumamoto University, 1-1-1 Honjo, Chuo-ku, Kumamoto 860-8556, Japan; 7 Core Research for Evolutional Science and Technology (CREST), Japan Science and Technology Agency, Tokyo, Japan

## Abstract

Bone metastasis of breast cancer cells is a major concern, as it causes increased morbidity and mortality in patients. Bone tissue-derived CXCL12 preferentially recruits breast cancer cells expressing CXCR4 to bone metastatic sites. Thus, understanding how CXCR4 expression is regulated in breast cancer cells could suggest approaches to decrease bone metastasis of breast tumor cells. Here, we show that tumor cell-derived angiopoietin-like protein 2 (ANGPTL2) increases responsiveness of breast cancer cells to CXCL12 by promoting up-regulation of CXCR4 in those cells. In addition, we used a xenograft mouse model established by intracardiac injection of tumor cells to show that *ANGPTL2* knockdown in breast cancer cells attenuates tumor cell responsiveness to CXCL12 by decreasing CXCR4 expression in those cells, thereby decreasing bone metastasis. Finally, we found that ANGPTL2 and CXCR4 expression levels within primary tumor tissues from breast cancer patients are positively correlated. We conclude that tumor cell-derived ANGPTL2 may increase bone metastasis by enhancing breast tumor cell responsiveness to CXCL12 signaling through up-regulation of tumor cell CXCR4 expression. These findings may suggest novel therapeutic approaches to treat metastatic breast cancer.

Breast cancer is the most common cancer type in women, and bone is the most common first site of metastasis in that cancer^1^,^2^,^3^. About 83% of patients with advanced breast cancer will develop bone metastases during the course of their disease^4^. The skeletal consequences of metastasis include pain, pathologic fractures, spinal cord and other nerve-compression syndromes, and life-threatening hypercalcemia, all of which cause increased morbidity and mortality^5^. Therefore, it is important to define mechanisms underlying bone metastasis of breast cancer cells.

The ligand of the CXCR4 chemokine receptor is the CXC chemokine stromal cell-derived factor 1 (SDF-1), also known as CXCL12^6^. Binding of CXCL12 to CXCR4 activates intracellular signaling associated with chemotaxis and cell survival^7^ and also functions in tumorigenesis and progression of various cancer subtypes^8^,^9^. CXCL12-activated CXCR4 signaling reportedly activates several signaling pathways, such as phosphatidylinositol 3-kinase (PI3K)/Akt and mitogen-activated protein kinase (MAPK), in various cell lines^7^ and regulates expression of matrix metalloproteinases (MMPs), which promote destruction of the extracellular matrix and are critical for metastasis^10^,^11^. ERK signaling induces MMP-13^12^,^13^, which cleaves collagen type I, which constitutes approximately 95% of bone collagen^14^. We previously reported that ANGPTL2 increases MMP expression and activity in osteosarcoma cells^15^. In breast cancer pathology, CXCL12 derived from various tissues, including bone tissue, preferentially recruits cancer cells expressing CXCR4 and promotes their metastasis to those tissues^16^,^17^, suggesting that CXCR4 suppression in breast cancer cells might be a strategy to decrease bone metastasis. However, molecular mechanisms underlying CXCR4 expression in tumor cells have not been fully clarified.

Angiopoietin-like proteins (ANGPTLs), which possess an N-terminal coiled-coil domain used for oligomerization and a C-terminal fibrinogen-like domain, are structurally similar to Tie-2 receptor ligands known as angiopoietins^18^. However, ANGPTLs do not bind to Tie2 or to its homologue Tie1 and thus function differently from angiopoietins^18^. ANGPTL2 is secreted primarily by adipose tissue in normal conditions^19^. We recently identified ANGPTL2 as a key mediator of chronic inflammation and associated diseases, such as obesity-related metabolic syndrome^19^, cardiovascular disease^20^,^21^, some autoimmune diseases^22^,^23^, carcinogenesis^24^,^25^ and tumor metastasis^15^,^26^. We also demonstrated that suppression of breast cancer cell-derived ANGPTL2 attenuated breast cancer metastasis to lung tissue *in vivo* using xenograft models created by implanting MDA-MB231 breast cancer cells into the mouse mammary fat pad^26^. We also found that serum ANGPTL2 levels in patients with metastatic breast cancer were significantly higher than those in patients with non-metastatic invasive ductal carcinoma^27^, suggesting that ANGPTL2 promotes breast cancer cell metastasis.

In the present study, we performed RNA sequence analysis of MDA-MB231 cells harboring *ANGPTL2* knockdown (MB231/miANGPTL2) and found that, relative to control (MB231/miLacZ) cells, CXCR4 expression significantly decreased, suggesting that ANGPTL2 contributes to CXCR4 expression in breast tumor cells. *In vitro* experiments revealed that MB231/miANGPTL2 attenuates breast tumor cell responsiveness to CXCL12 stimulation by decreasing CXCR4 expression in those cells. We also found that ETS1-dependent transcription was important for ANGPTL2-induced CXCR4 expression and that ANGPTL2 increased breast tumor cell invasiveness by activating ERK and MMP-13 expression. Using a xenograft mouse model established by intracardiac injection of tumor cells, we found that mice injected with MB231/miANGPTL2 cells showed significantly decreased bone metastasis and prolonged survival relative to controls. Finally, we observed a positive correlation of ANGPTL2 and CXCR4 expression in primary tumor tissues from breast cancer patients. These findings suggest that tumor cell-derived ANGPTL2 may increase bone metastasis by enhancing breast tumor cell responsiveness to CXCL12 signaling through up-regulation of tumor cell CXCR4 expression.

## Results

### ANGPTL2 suppression in MDA-MB231 cells attenuates CXCL12-activated CXCR4 signaling and expression

**Figure 1:**
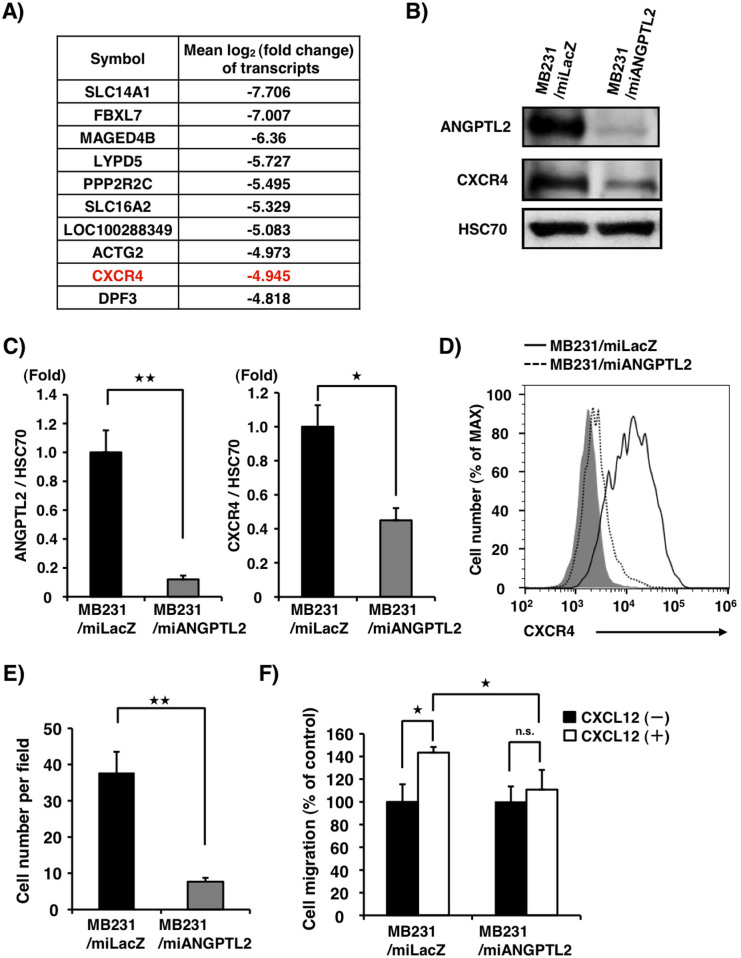
ANGPTL2 regulates CXCR4 induction in MDA-MB231 cells. (A) Ranking of the top 10 down-regulated genes in MB231 cells following *ANGPTL2* knockdown, based on RNA sequencing analysis. Data are mean log_2_ value of fold-change relative to control MB231/miLacZ cells. (B) Representative image showing western blot analysis of ANGPTL2 and CXCR4 in MB231/miLacZ and MB231/miANGPTL2 cells. Full-length blots are presented in Supplementary Fig. S2A. (C) Quantitative protein levels of ANGPTL2 and CXCR4 relative to HSC70. Data from MB231/miLacZ was set at 1. Data are means ± SEM from three experiments; ^★^*P* < 0.05, ^★★^*P* < 0.01 (unpaired two-tailed Student's *t*-test). (D) CXCR4 cell surface expression in MB231/miANGPTL2 and MB231/miLacZ cells based on FACS analysis. Gray area indicates isotype control antibody. MB231/miANGPTL2 cells, dotted line; MB231/miLacZ cells, solid line. (E) Transwell migration assay showing MB231/miLacZ or MB231/miANGPTL2 cell migration in the absence of CXCL12 stimulation. Data are means ± SEM from five experiments; ^★★^*P* < 0.01 (unpaired two-tailed Student's *t*-test). (F) Relative migration of MB231/miANGPTL2 or MB231/miLacZ cells in a transwell assay in the presence of CXCL12 (100 ng/ml). Data from the control (absence of CXCL12 stimulation, respectively) was set at 100. Data are means ± SEM from five experiments; ^★^*P* < 0.05 (unpaired two-tailed Student's *t*-test).

Our previous findings in an orthotopic implantation model showed that *ANGPTL2* knockdown in breast cancer cells reduces metastasis to distant tissues, such as lung^26^. To determine what factors downstream of ANGPTL2 might promote metastasis, we compared transcripts in *ANGPTL2* knockdown human breast tumor MB231 cells to those in control MB231 cells using an RNA sequencing strategy. To do so, we generated both MB231/miANGPTL2 and control *LacZ* knockdown (MB231/miLacZ) lines using the Invitrogen BLOCK-iT miR RNAi system^15^, as previously reported^26^. We observed twenty transcripts whose expression was markedly altered (10 upregulated and 10 downregulated) in MB231/miAngptl2 compared to control cells (Fig. 1A, Supplementary Fig. S1). Among them, we focused on the chemokine receptor C-X-C chemokine receptor type 4 (CXCR4), whose expression was decreased in the MB231/miANGPTL2 line. CXCR4 was of particular interest because breast cancer cells expressing CXCR4 reportedly move to secondary sites in lung, liver, bone marrow or lymph nodes where the CXCR4 ligand, the chemokine CXCL12, is produced in at high levels^16^,^28^. In addition, silencing of CXCR4 in breast cancer cells reportedly reduces metastasis in a mouse xenograft model^29^.

Western blot analysis confirmed that CXCR4 protein levels were significantly lower in MB231/miANGPTL2 compared to MB231/miLacZ cells (Fig. 1B and 1C, Supplementary Fig. S2A and S2B). When we assessed cell surface CXCR4 expression by FACS analysis, we found that MB231/miANGPTL2 cells expressed low CXCR4 levels compared to control MB231/miLacZ cells (Fig. 1D). By contrast, CXCL12 expression in both lines was comparable (Supplementary Fig. S2C).

To compare motility of MB231/miANGPTL2 and MB231/miLacZ cells, we used a trans-well assay in the presence or absence of exogenous CXCL12. In the absence of CXCL12, MB231/miANGPTL2 cells showed lower motility than did control MB231/miLacZ cells (Fig. 1E). In addition, MB231/miANGPTL2 cells treated with CXCL12 showed lower motility than did similarly-treated control cells (Fig. 1F, Supplementary Fig. S3), suggesting that ANGPTL2 loss in these cells decreases their responsiveness to CXCL12, most likely due to downregulation of CXCR4.

### ANGPTL2 effects on tumor invasivity are accompanied by CXCL12-dependent ERK1/2 signaling and MMP-13 expression

**Figure 2:**
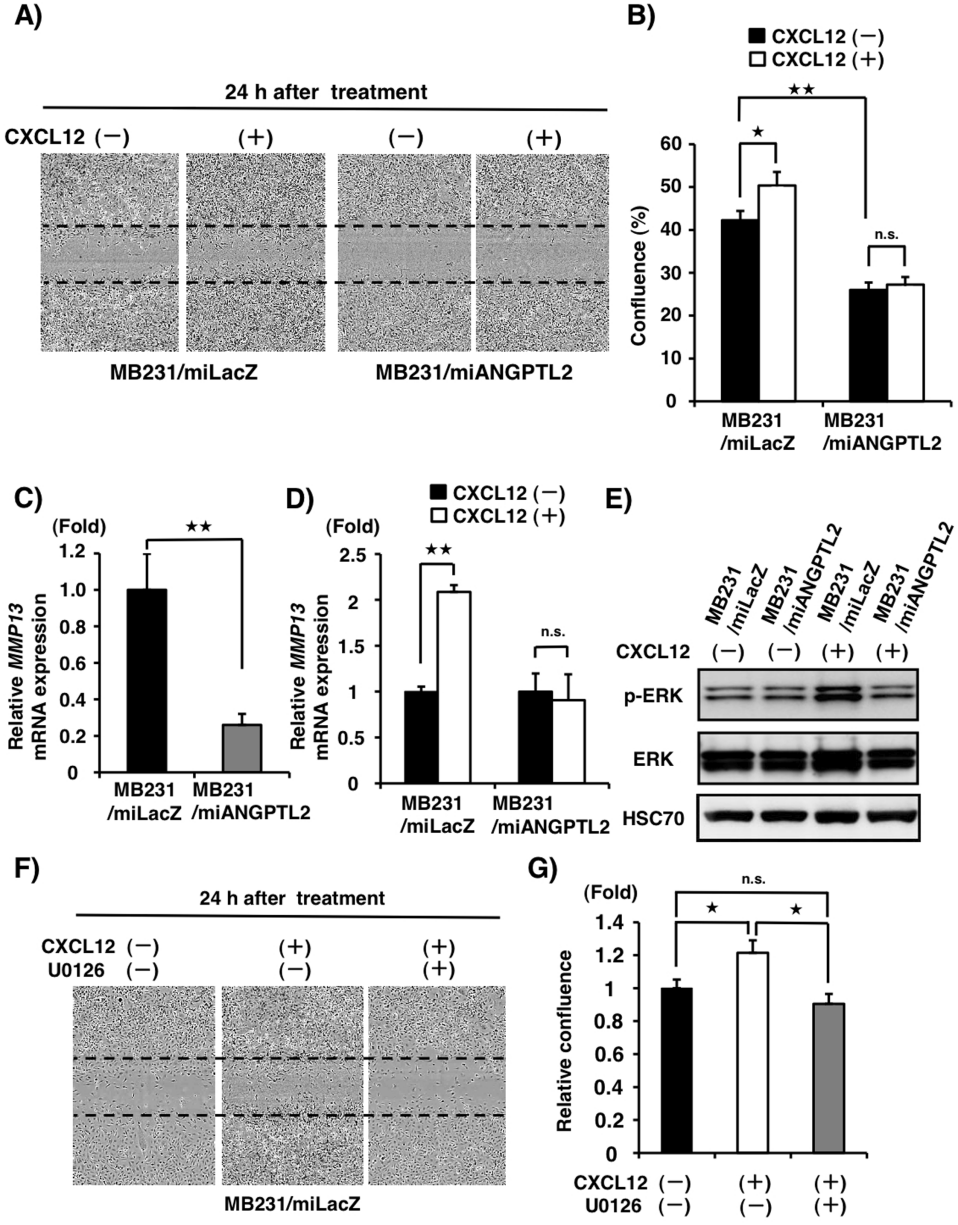
ANGPTL2 enhances CXCL12-activated and MMP-13-dependent cell invasion accompanied by ERK1/2 activation. (A) Invasion assay of MB231/miANGPTL2 and MB231/miLacZ cells on type I collagen-coated plates. Representative images of invasion 24 h after treatment with or without CXCL12. Time courses are shown in Supplementary Fig. S4. (B) Quantitative assay of invasivity by MB231/miANGPTL2 or MB231/miLacZ cells as indicated by the rate of confluence of a scratched area 24 h after treatment with or without CXCL12. Data are means ± SEM from five experiments; ^★^*P* < 0.05, ^★★^*P* < 0.01 (unpaired two-tailed Student's *t*-test). (C) Relative *MMP-13* expression in MB231/miANGPTL2 or MB231/miLacZ cells. Data from MB231/miLacZ was set at 1. Data are means ± SEM from three experiments; ^★★^*P* < 0.01(unpaired two-tailed Student's *t* test). (D) Relative *MMP-13* expression in MB231/miANGPTL2 and MB231/miLacZ cells 8 h after CXCL12 treatment. Data from control (absence of CXCL12 stimulation, respectively) was set at 1. Data are means ± SEM from three experiments; ^★★^*P* < 0.01(unpaired two-tailed Student's *t*-test). (E) Representative immunoblot of indicated lines showing phosphorylated ERK1/2 (p-ERK), total ERK1/2 and HSC70 10 min after CXCL12 treatment. Full-length blots are presented in Supplementary Fig. S5. Related time courses are presented in Supplementary Fig. S6. (F) Invasion assay of MB231/miLacZ cells on type I collagen-coated plates. Representative image showing invasion in response to CXCL12 with or without the MKK inhibitor U0126, 24 h after treatment. (G) Relative confluence of cells subjected to the invasion assay, 24 h after treatment. Occupation area is calculated based on control (without CXCL12 and U0126) data, which was set at 1. Data are means ± SEM from five experiments; ^★^*P* < 0.05 (unpaired two-tailed Student's *t*-test).

To determine whether attenuated CXCL12-dependent CXCR4 signaling in *ANGPTL2* knockdown MB231 cells alters their invasivity, we compared CXCL12-accelerated, collagenolysis-dependent tumor cell invasivity in MB231/miANGPTL2 versus control MB231/miLacZ cells using wound healing assays on type I collagen-coated plates. In the absence of CXCL12 treatment, MB231/miLacZ cells showed greater invasivity than did MB231/miANGPTL2 cells (Fig. 2A and 2B, Supplementary Fig. S4). In addition, MB231/miLacZ cells treated with CXCL12 also showed greater invasivity than did MB231/miANGPTL2 cells (Fig. 2A and 2B, Supplementary Fig. S4).

Next we used quantitative real-time PCR to compare the effect of CXCL12 on MMP-13 expression in MB231/miANGPTL2 and control MB231/miLacZ cells. In the absence of CXCL12 treatment, MMP-13 expression was lower in MB231/miANGPTL2 compared to MB231/miLacZ cells (Fig. 2C). Treatment of cells with CXCL12 for 8 hours increased MMP-13 expression in MB231/miLacZ controls but not in MB231/miANGPTL2 cells (Fig. 2D). Western blot analysis of CXCL12-treated cells revealed transient ERK1/2 activation in MB231/miLacZ cells but not in MB231/miANGPTL2 cells (Fig. 2E, Supplementary Fig. S5 and S6), suggesting that ERK1/2 activation underlies induction of MMP-13 promoted by CXCL12. To further assess this possibility, we undertook invasion assays of control MB231/miLacZ cells treated with various combinations of CXCL12 or the MKK inhibitor U0126. Quantitative analysis of a given invasion area revealed that the area occupied by invasive MB231/miLacZ cells seen following CXCL12 treatment significantly decreased when the assay was conducted in the presence of U0126 (Fig. 2F and G). These findings suggest that *ANGPTL2* knockdown in MDA-MB231 cells antagonizes CXCL12-dependent ERK1/2 activation and reduces MMP-13-induction required for tumor invasive activity.

### ANGPTL2 enhances both CXCR4 and ETS1 expression in MDA-MB231 cells

**Figure 3:**
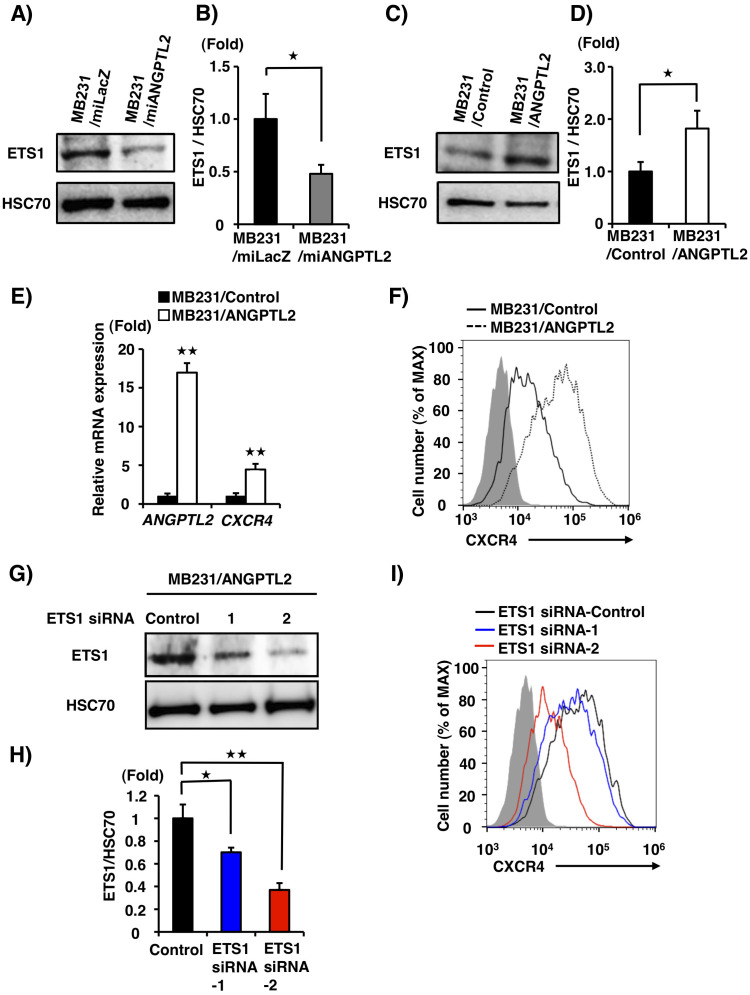
ANGPTL2 enhances CXCR4 and ETS1 expression in MDA-MB231 cells. (A) Representative image showing immunoblot of ETS1 and HSC70 in MB231/miANGPTL2 and MB231/miLacZ cells. Full-length blots are presented in Supplementary Fig. S7A. (B) Quantitative ETS1 protein levels relative to HSC70. Data from MB231/miLacZ was set at 1. Data are means ± SEM from three experiments; ^★^*P* < 0.05 (unpaired two-tailed Student's *t*-test). (C) Representative immunoblot of ETS1 and HSC70 in MB231/Control and MB231/ANGPTL2 cells. Full-length blots are shown in Supplementary Fig. S7D. (D) Quantitative ETS1 protein levels relative to HSC70. Data from MB231/Control was set at 1. Data are means ± SEM from three experiments; ^★^*P* < 0.05 (unpaired two-tailed Student's *t*-test). (E) Relative ANGPTL2 and CXCR4 expression in MB231/ANGPTL2 cells. Data are means ± SEM from three experiments; ^★★^*P* < 0.01(unpaired two-tailed Student's *t*-test). (F) CXCR4 cell surface expression in MB231/ANGPTL2 and MB231/Control cells based on flow cytometry. Gray shaded area represents isotype control antibody group. MB231/ANGPTL2 cells, dotted-line; MB231/Control cells, solid line. (G) Representative image and analysis of immunoblotting for ETS1 relative to HSC70 in MB231/ANGPTL2 cells transduced with ETS1 siRNA. Full-length blots are presented in Supplementary Fig. S8. (H) Quantitative ETS1 protein levels relative to HSC70. Control siRNA cells, black bar; ETS1 siRNA-1 cells, blue; and ETS1 siRNA-2 cells, red. Data from Control was set at 1. Data are means ± SEM from three experiments; ^★^*P* < 0.05, ^★★^*P* < 0.01 (unpaired two-tailed Student's *t*-test) (I) CXCR4 cell surface expression in Control siRNA, ETS1 siRNA-1, and ETS1 siRNA-2 cells based on flow cytometry. Gray shaded area represents isotype control antibody group. Control siRNA cells, black line; ETS1 siRNA-1, blue; and ETS1 siRNA-2, red.

We next examined the molecular basis for enhanced CXCR4 expression by ANGPTL2. The transcription factor ETS1 (for Erythroblastosis virus E26 oncogene homolog 1) reportedly regulates CXCR4 expression in tumor cells, as do other transcription factors^30^,^31^,^32^,^33^,^34^,^35^,^36^. When we examined ETS1 expression by western blotting, we found that it was significantly decreased in MB231/miANGPTL2 compared to control MB231/miLacZ cells (Fig. 3A and 3B, Supplementary Fig. S7A). Next, we asked whether increasing ANGPTL2 expression in MDA-MB231 cells promoted ETS1 expression. To do so, we generated MDA-MB231 cell lines that constitutively express *ANGPTL2* (MB231/ANGPTL2) and control lines expressing vector only (MB231/Control) (Supplementary Fig. S7B). Unlike *ANGPTL2* knockdown cells, ETS1 expression increased in MB231/ANGPTL2 compared to MB231/Control cells (Fig. 3C and 3D, Supplementary Fig. S7D). Furthermore, *CXCR4* mRNA expression significantly increased in MB231/ANGPTL2 compared to MB231/Control cells (Fig. 3E). FACS analysis indicated that MB231/ANGPTL2 cells expressed higher levels of cell surface CXCR4 than did MB231/Control cells (Fig. 3F). In contrast, CXCL12 expression in these lines was comparable (Supplementary Fig. S7C).

Next we examined cell surface CXCR4 expression in MB231/ANGPTL2 cells after *ETS1* knockdown using ETS1 siRNA (Fig. 3G and 3H, Supplementary Fig. S8). FACS analysis revealed reduced cell surface CXCR4 expression in *ETS1* knockdown versus control cells (Fig. 3I), suggesting a link between ANGPTL2-induced CXCR4 expression and ETS1 expression.

To investigate whether ANGPTL2 increases CXCR4 expression in other breast cancer cells, we generated T47-D lines that constitutively express ANGPTL2 (T47-D/ANGPTL2) and control vector-only lines (T47-D/Control) (Supplementary Fig. S9A). As anticipated, we found that *CXCR4* and *ETS1* mRNA expression significantly increased in T47-D/ANGPTL2 compared to T47-D/Control cells (Supplementary Fig. S9B and S9C). FACS analysis indicated that T47-D/ANGPTL2 cells expressed higher levels of cell surface CXCR4 than did T47-D/Control cells (Supplementary Fig. S9D). These results suggest that ANGPTL2 may enhance CXCR4 expression in breast cancer cells by increasing ETS1 expression.

### Suppression of tumor cell-derived ANGPTL2 decreases bone metastasis in an intracardiac inoculation model

**Figure 4:**
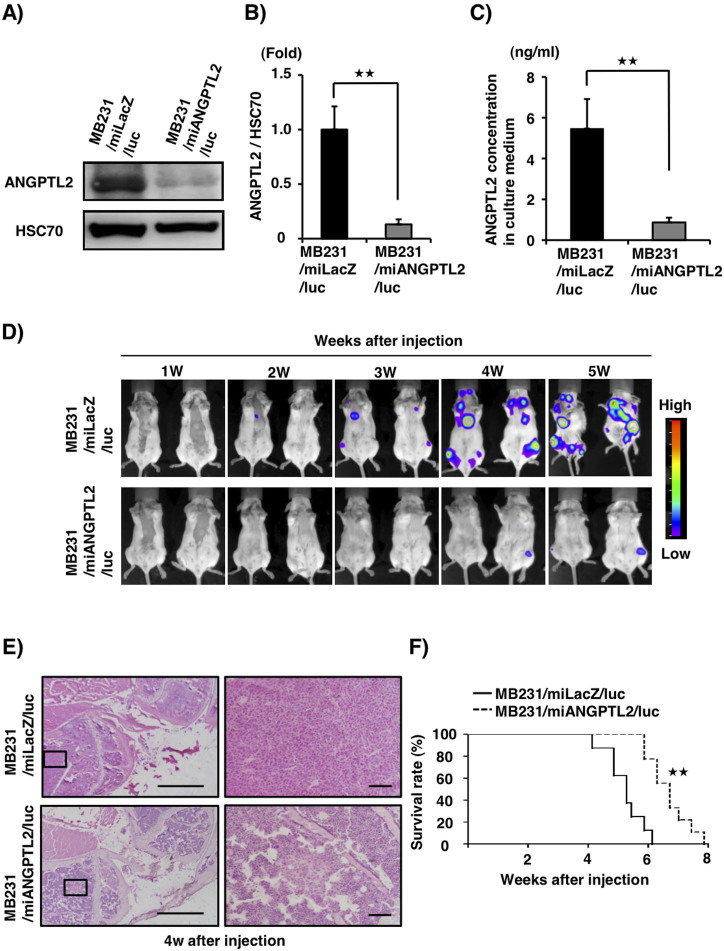
Suppression of tumor cell-derived ANGPTL2 decreases bone metastasis in an intracardiac inoculation model. (A) Representative image showing western blot analysis of ANGPTL2 in MB231/miLacZ/luc and MB231/miANGPTL2/luc cells. Full-length blots are presented in Supplementary Fig. S10. (B) Quantitative ANGPTL2 protein levels relative to HSC70. Data from MB231/miLacZ/luc was set at 1. Data are means ± SEM from three experiments; ^★★^*P* < 0.01 (unpaired two-tailed Student's *t*-test). (C) Comparison of ANGPTL2 levels in culture medium of indicated cells. Data are means ± SEM from three experiments; ^★★^*P* < 0.01(unpaired two-tailed Student's *t*-test). (D) Representative image showing bioluminescence signals in xenografted mice. MB231/miLacZ/luc or MB231/miANGPTL2/luc cells were injected into the left cardiac ventricle of immunodeficient mice (n = 8). At indicated times after xenografting, bioluminescence signals were captured. Images for weeks 1–5 are displayed on the same scale. (E) Representative microscopy images of tumor cells metastasized to tibial bone (as indicated by H&E staining) 4 weeks after injection with MB231/miLacZ/luc (top) or MB231/miANGPTL2/luc (bottom) cells. Right panels are magnifications of squares in left panels. Left image scale bar = 1.0 mm; right image scale bar = 100 μm. (F) Kaplan-Meier survival curves of mice bearing tumors derived from MB231/miLacZ (n = 8) or MB231/miANGPTL2 (n = 9) cells. ^★★^*P* < 0.01 (log-rank test).

Since breast cancer cells expressing CXCR4 tend to move toward regions of high CXCL12 expression^16^,^28^, we asked whether *ANGPTL2* knockdown and concomitant decreases in CXCR4 expression would alter the metastatic potential of MDA-MB231 cells. To do so, we established *ANGPTL2* knockdown and control cells harboring a luciferase expression vector (MB231/miANGPTL2/luc and MB231/miLacZ/luc, respectively), as we previously reported^26^. Cellular ANGPTL2 protein levels as well as ANGPTL2 levels in the culture medium were significantly decreased in knockdown compared to control cell lines (Fig. 4A–4C, Supplementary Fig. S10). We observed no differences in proliferation in these lines under normoxia or hypoxia in vitro (Supplementary Fig. S11A). Knockdown and control lines also exhibited no difference in primary tumor size in an orthotopic in vivo implantation model (Supplementary Fig. S11B). To investigate whether tumor cell-derived ANGPTL2 enhances the ability of cells to colonize a metastatic site, we injected MB231/miANGPTL2/luc or MB231/miLacZ/luc cells into the left cardiac ventricle of immunodeficient mice and assessed metastasis. Three weeks after injection of control MB231/miLacZ/luc cells, we detected bone metastasis. However, bone metastasis was detected one week later (at four weeks) in MB231/miANGPTL2/luc-injected mice (Fig. 4D). At 5 weeks after treatment, metastatic activity was greater in MB231/miLacZ- compared to MB231/miANGPTL2/luc-injected mice. When we observed metastatic lesions using H&E staining, we observed significantly decreased bone colonization in MB231/miANGPTL2/luc- compared to MB231/miLacZ/luc-injected mice: the bone-marrow cavity of MB231/miLacZ/luc-injected mice was almost filled with tumor cells by four weeks after injection. (Fig. 4E, Supplementary Fig. S12). Finally, survival of MB231/miANGPTL2-injected mice was prolonged relative to MB231/miLacZ-injected mice (Fig. 4F).

To examine whether *CXCR4* knockdown in ANGPTL2-expressing cells decreased bone metastasis, we first established ANGPTL2-overexpressing and control lines each harboring a luciferase expression vector (MB231/ANGPTL2/luc and MB231/Control/luc, respectively). ANGPTL2 protein levels in the culture medium were significantly higher in MB231/ANGPTL2/luc compared to MB231/Control/luc cells (Supplementary Fig. S13A). We then injected both lines into the left cardiac ventricle of immunodeficient mice and assessed metastasis in each group. We detected greater metastatic activity by four weeks after injection of MB231/ANGPTL2/luc compared to MB231/Control/luc cells, and survival of MB231/ANGPTL2/Luc-injected mice was shorter relative to MB231/Control-injected mice (Supplementary Fig. S13B and S13C). Next, we established *CXCR4* knockdown ANGPTL2-overexpressing and control cells (MB231/ANGPTL2/miCXCR4/luc and MB231/ANGPTL2/miLacZ/luc, respectively). *CXCR4* mRNA and cell surface protein expression was significantly decreased in MB231/ANGPTL2/miCXCR4/luc compared to MB231/ANGPTL2/miLacZ/luc cells (Supplementary Fig. S14A and S14B). When we injected MB231/ANGPTL2/miCXCR4/luc or MB231/ANGPTL2/miLacZ/luc cells into the left cardiac ventricle of immunodeficient mice to assess metastasis, we detected less metastatic activity by 3 weeks after injection of MB231/ANGPTL2/miCXCR4/luc-injected compared to MB231/Control-injected mice (Supplementary Fig. S14C and S14D).

### ANGPTL2 expression in primary tumor tissues correlates with CXCR4 expression, lymph node metastasis and breast cancer progression

**Figure 5:**
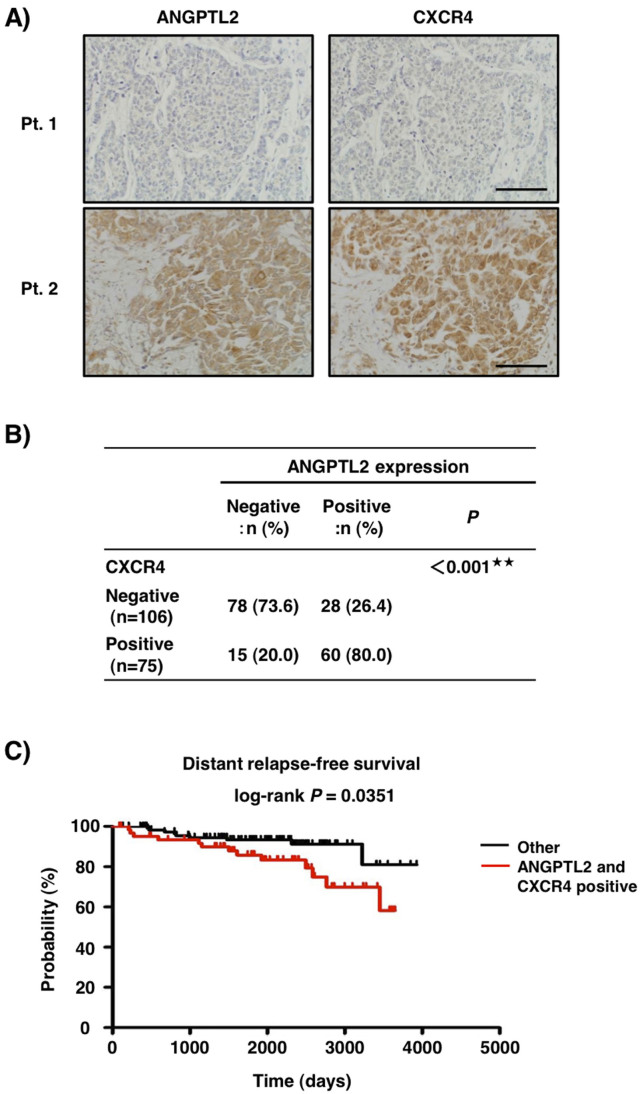
ANGPTL2 expression at primary tumor sites correlates with CXCR4 expression. (A) ANGPTL2 immunostaining within a primary tumor as seen in tissue specimens derived from patients. Representative images of ANGPTL2-negative and CXCR4-negative (Patient (Pt.) 1) or ANGPTL2-positive and CXCR4-positive (Pt.2) specimens. Scale bar = 100 μm. (B) Distribution of ANGPTL2 and CXCR4 staining in breast cancer specimens. ^★★^*P* < 0.001(Fisher's exact test). (C) Cohort of probability of distant relapse-free survival in both ANGPTL2- and CXCR4-positive (n = 60) and other (ANGPTL2-negative or CXCR4-negative) (n = 121) groups (*P* = 0.0351 by log-rank test).

Next, we analyzed a potential relationship between ANGPTL2 and CXCR4 expression in primary tumors from 181 breast cancer patients (Fig. 5A). Patients were divided into two groups based on percentage of ANGPTL2-positive tumor cells at the primary tumor site: the positive group was defined as showing ≥50% ANGPTL2-positive tumor cells, while the negative group showed <50%. This analysis showed that ANGPTL2 expression was positively correlated with high CXCR4 expression in specimens (Fig. 5B). ANGPTL2 expression at the primary tumor site was also significantly correlated with tumor size (T-factor), positivity for lymph node metastasis, and severity of breast cancer stage (*P* < 0.001; Supplementary Table 2). CXCR4 expression at the primary tumor site was correlated only with positivity for lymph node metastasis (*P* < 0.01; Supplementary Table 3). When we asked whether ANGPTL2 or CXCR4 levels correlated with patient outcome, we found that patients in the ANGPTL2-positive group showed a shortened period of distant relapse-free survival (DRFS) after surgery relative to the negative group (Supplementary Fig. S17A). Patients in the CXCR4-positive and -negative groups showed comparable DRFS periods (Supplementary Fig. S17B). However, patients in the group positive for both ANGPTL2 and CXCR4 showed a shortened DRFS period compared to the other patients group (Fig. 5C). Next, we analyzed the relationship among ANGPTL2, CXCR4 and MMP-13 expression in primary tumors. Both ANGPTL2 and CXCR4 expression levels were positively correlated with high MMP-13 expression in breast cancer patient specimens (Supplementary Fig. S15 and S16). Patients in MMP-13 positive and -negative groups showed comparable DRFS periods (Supplementary Fig. S17C). Overall, these findings suggest that ANGPTL2 may promote breast cancer progression, possibly by activating CXCR4 and MMP-13.

We next examined how ANGPTL2 might induce CXCR4. We previously reported that ANGPTL2 binds to the integrin α5β1 receptor, and others have reported that ANGPTL2 binds to leukocyte Ig-like receptor B2 (LILRB2) to promote hematopoietic stem cell expansion^37^. Thus, we asked whether MDA-MB231 cells express either candidate receptor. Flow cytometry analysis showed that LILRB2 was not expressed in MDA-MB231 cells (Supplementary Fig. S18A), while integrin α5β1 was (Supplementary Fig. S18B). When we asked whether treatment of MDA-MB231 cells with a neutralizing antibody for integrin α5β1 could block CXCR4 induction, we observed partial but significant suppression of CXCR4 induction by that antibody (Supplementary Fig. S18C).

## Discussion

**Figure 6:**
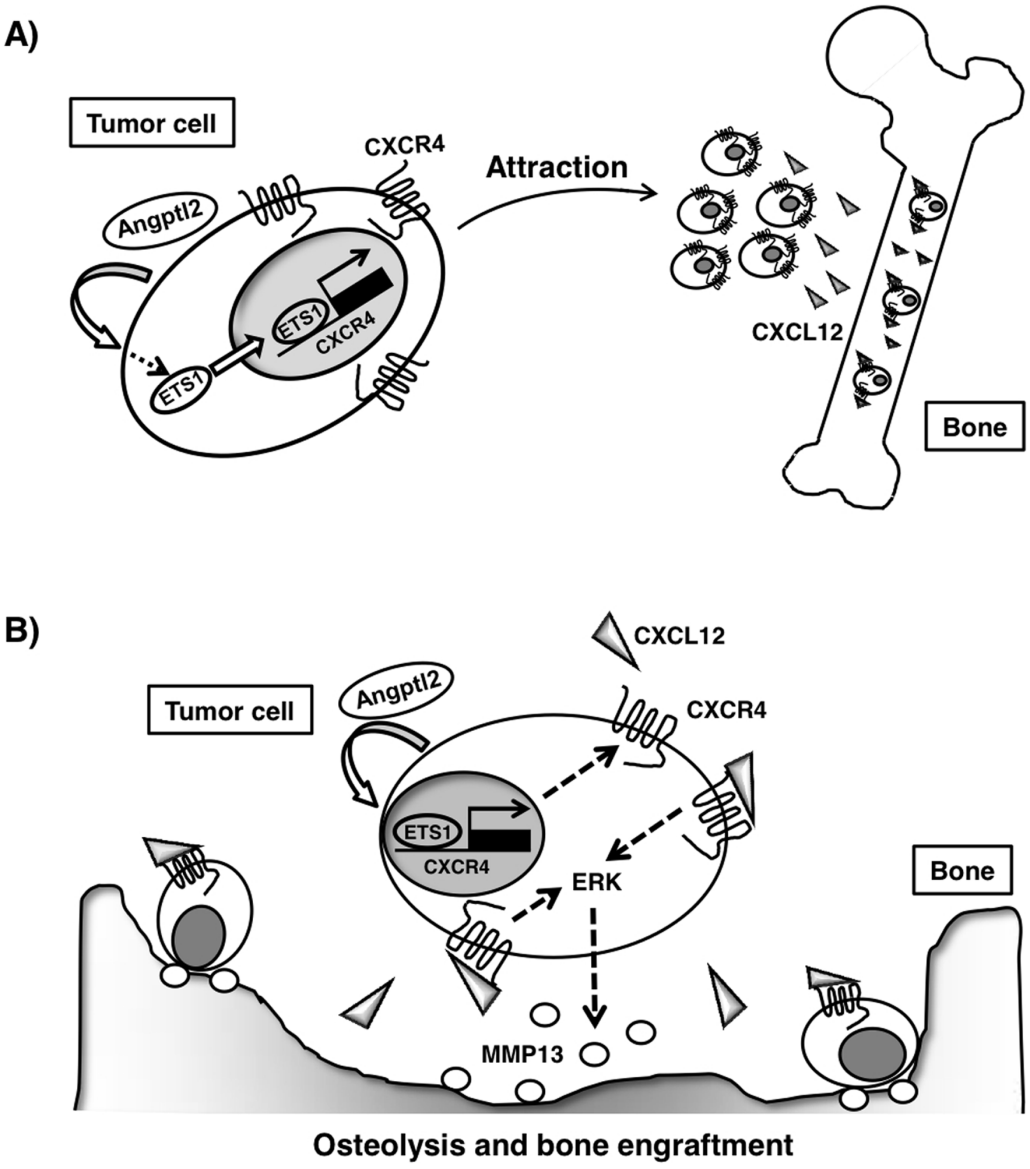
Mechanistic model of crosstalk between ANGPTL2 and CXCL12-activated CXCR4 signaling in breast cancer metastasis to bone. (A) Tumor cell-derived ANGPTL2 increases CXCR4 expression in an autocrine manner, potentially through ETS1 up-regulation, resulting in increased metastasis of tumor cells to bone, a region of high expression of the CXCR4 ligand CXCL12. (B) In bone, CXCL12-activated CXCR4 signaling increases MMP-13 expression and accelerates osteolysis and bone engraftment.

Here we demonstrate that breast cancer cell-derived ANGPTL2 may play an important role in metastasis of breast cancer cells to bone. Several lines of evidence suggest this possibility. First, we found that ANGPTL2 enhances both CXCR4 and ETS1 expression, suggesting that these outcomes are linked. Our findings also suggest that ANGPTL2 increases bone metastasis by up-regulating CXCR4 in primary tumor cells, thereby enhancing their responsiveness to bone tissue-secreted CXCL12 (Fig. 6A). Furthermore, in bone tissue, CXCL12-activated CXCR4 signaling promoted by ANGPTL2 accelerated osteolysis and bone engraftment most likely by increasing MMP-13 activity (Fig. 6B). Finally, ANGPTL2 expression was positively correlated with CXCR4 expression levels in primary tumor tissue from breast cancer patients.

CXCL12 signaling through CXCR4 plays important physiological roles, as in hematopoietic stem cell homing to the bone marrow niche^38^. CXCL12 signaling through CXCR4 also plays critical roles in cancer metastasis. In breast cancer, CXCL12 derived from various tissues recruits breast tumor cells that express CXCR4 by increasing their chemotaxis to regions of high CXCL12 expression, such as lymph nodes, lung, liver, and bone tissues^16^. Thus CXCR4 down-regulation in breast cancer cells could decrease their metastasis. In this study, we showed that ANGPTL2 significantly up-regulated CXCR4 expression in breast cancer cells, which likely enhances their responsiveness to CXCL12. We previously reported that ANGPTL2-expressing breast cancer or osteosarcoma cells metastasized to lung^15^,^26^, where CXCL12 expression is high. Therefore, ANGPTL2 activity is likely not limited to bone. However, in this study, bone metastasis by ANGPTL2-expressing cells is often observed in the mouse intracardiac inoculation model. As yet uncharacterized mechanisms that we will address in future studies may govern a preference for bone metastasis.

Molecular mechanisms underlying CXCR4 expression have not been fully clarified, although factors such as HIF1α, NF-κB, and TGF-β reportedly regulate CXCR4 expression^39^. In particular, NF-κB induces CXCR4 expression in tumor cells^32^, and we observed that cell surface CXCR4 expression in breast cancer cells is partially reduced by treatment with an NF-κB inhibitor (unpublished results). This finding suggests the possibility that NF-κB is also activated by ANGPTL2, a conclusion consistent with our previous report that ANGPTL2 activates NF-κB-dependent pro-inflammatory signaling^19^,^23^.

The present study suggests that ETS1-dependent transcriptional regulation is important for ANGPTL2-induced CXCR4 expression. ETS1 activity is also reportedly important for transformation of endothelial cell phenotypes from quiescent to angiogenic^40^ and for regulation of angiogenesis by pro-angiogenic factors, such as VEGF^41^, angiotensin II^42^ or TGF-β^43^. Therefore, tumor cell-derived ANGPTL2 may up-regulate ETS1-dependent activation of pro-angiogenic genes, promoting tumor angiogenesis. ANGPTL2 also exhibits direct pro-angiogenic activity^24^. Taken together with these findings, ANGPTL2 likely plays both direct and indirect roles in tumor angiogenesis in the case of breast cancers, and these effects may underlie enhanced bone metastasis.

We also found that increased MMP-13 expression seen following CXCL12 treatment was significantly attenuated in *ANGPTL2* knockdown cells. Many reports indicate that elevated circulating MMP-13 levels are associated with progression and metastasis of various cancers and specifically correlate with decreased overall patient survival and increased lymph node metastasis in breast cancer^44^, increased bone metastasis in renal cell carcinoma^45^, and poor prognosis of non-small cell lung and colorectal cancers^46^,^47^. MMP-13 cleaves type I collagen, which constitutes almost all collagen found in bone. In breast cancer pathology, MMP-13-selective inhibitors show effective therapeutic effects against cancer-induced bone osteolysis^48^,^49^. Recently, we reported that ANGPTL2 enhances tumor cell invasion by increasing expression of MMP-1, MMP-9, and MMP-13 In osteosarcoma cells^15^,^21^. Thus, ANGPTL2 secretion from cancer cells likely contributes to MMP activity in those cells.

In summary, here we show that breast cancer cell-derived ANGPTL2 may play important roles in bone metastasis by enhancing responsiveness of breast cancer cells to bone tissue-secreted CXCL12 through up-regulated tumor cell CXCR4 expression. Furthermore, our findings suggest that in bone tissue, CXCL12-activated CXCR4 signaling in breast cancer cells induced by ANGPTL2 also accelerates osteolysis and bone engraftment. These studies could form the basis of new therapeutic strategies to antagonize bone metastasis of breast cancer cells.

## Methods

### Cell culture

Human breast adenocarcinoma cell lines MDA-MB231 and T47-D were purchased from the American Type Culture Collection (ATCC). Cells were confirmed to be identical to cells registered with ATCC by comparison with the database of the JCRB Cell Bank. MDA-MB231 cells were cultured in Leibovitz's L15 (Wako, Osaka, Japan) medium supplemented with 10% FCS under 100% air without CO_2_, and T47-D cells were cultured in RPMI-1640 (Wako) medium supplemented with 10% FCS under 5% CO_2_/95% air.

### Proliferation assay

Proliferation assays were performed using the Cell Counting Kit-8 (Dojindo, Kumamoto, Japan) in normoxia (under 100% air without CO_2_) and hypoxia (1% O_2_ without CO_2_) conditions. 100 μl cell suspensions (5 × 10^4^/ml) with 10% FCS medium was added to a 96-well plate and incubated for 6 hour at 37°C. Then, 10 μl CCK-8 reagent was added to each well, cells were incubated 2 hours at 37°C, and optical density at 450 nm was measured. Similar assays were performed after 24, 48, and 72 hours. The growth rate was expressed as a value relative to hour 0.

### MicroRNAi silencing of ANGPTL2 expression

MDA-MB231 cells were transfected with ANGPTL2-specific small interfering RNA vectors and control vectors^26^ using Lipofectamine 2000 (Invitrogen, Carlsbad, CA, USA) according to the manufacturer's protocol. MicroRNAi constructs were made using the BLOCK-iT Pol II miR RNAi expression vector kit, according to the manufacturer's instructions (Invitrogen). For microRNA synthesis, a single stranded-DNA oligo encoding a pre-miRNA and a strand encoding its complement were designed using the Invitrogen RNAi Designer website: ANGPTL2 (top) 5′-GATCCGGAGCCCTCACTCTCCAGATTCGAAGCTTGGAATCTGGAGAGTGAGGGCTCTTTTTTGGAAG-3′ and ANGPTL2 (bottom) 5′-GCCTCGGGAGTGAGAGGTCTAAGCTTCGAACCTTAGACCTCTCACTCCCGAGAAAAAACCTTCTTAA-3′, CXCR4 (top) 5′-TGCTGTAAACATCACAACTGGACTCGGTTTTGGCCACTGACTGACCGAGTCCATGTGATGTTTA-3′ and CXCR4 (bottom) 5′-CCTGTAAACATCACATGGACTCGGTCAGTCAGTGGCCAAAACCGAGTCCAGTTGTGATGTTTAC-3′. Stable lines were selected in 20 μg/ml Blasticidin (Invitrogen).

### RNA sequencing

Sample libraries established from MB231/miANGPTL2 and MB231/miLacZ cells were prepared using a TruSeq RNA Sample Prep Kit (Illumina, San Diego, CA, USA). Sequencing runs were performed on an Illumina Genome Analyzer IIx (Illumina). 38 bp reads were mapped to the human genome (hg19 from the UCSC genome browser database) using TopHat v2.0.0.^50^. Only reads with a Phred quality score ≥ 25 were analyzed. The BEDtools package^51^ was used to filter rRNA and tRNA, with rRNA and tRNA annotations downloaded from the UCSC table browser.

To evaluate differential expression, expression data was normalized, and gene annotations were added using RegionMiner with Genomatix Genome Analyzer (Genomatix, Munich, Germany) software. The Normalized Expression value (NE-value) was based on the following formula:
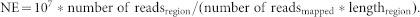
52

To compile NE-value information for an individual gene, mean NE-values were calculated from all gene transcripts, as were log2-transformed fold-changes between MB231/miANGPTL2 and MB231/miLacZ cells. RNA-seq data were deposited in the DDBJ Sequence Read Archive (DRA). Accession number: DRA002859.

### Quantitative real-time PCR

Total RNA was extracted from cells using TRIzol reagent (Invitrogen). Deoxyribonuclease-treated RNA was reverse-transcribed with a PrimerScript RT Reagent Kit (Takara Bio, Otsu, Shiga, Japan). PCR was performed using SYBR Premix Ex Taq II (Takara Bio). Oligonucleotides used for PCR are listed in Supplementary Table 1. PCR products were analyzed with a Thermal Cycler Dice Real Time system (Takara Bio), and relative transcript abundance was normalized to that of RPS18.

### Flow cytometry (FACS)

Subconfluent cells were detached using Accutase Solution (Wako) at 37°C. Cells were incubated with rabbit anti-human antibody against PE-CXCR4 (BioLegend, San Diego, CA, USA), α5β1 (Millipore, Temecula, CA, USA) and PE-LILRB2 (Beckman Coulter, Brea, CA, USA) for 30 min at 4°C, washed with PBS and then analyzed by flow FACSCalibur (BD Biosciences, Franklin Lakes, NJ, USA). Subsequent data analysis was done using FlowJo software (Treestar, Ashland, OR, USA).

### Transwell migration assay

Assays were performed using a Costar Transwell Permeable Support system with 8.0 μm pore size. In brief, 5 × 10^4^ cells were suspended in 1% FCS media and placed in the upper chamber of a transwell plate without ligand, while the lower chamber contained 1% FCS media plus CXCL12 at a final concentration of 50, 100 or 200 ng/ml (R&D System, Minneapolis, MN, USA). After 18 h incubation at 37°C, cells on the upper surface of the filter were removed by wiping with cotton swab, and cells that had migrated to the lower side were fixed with formalin and stained with Giemsa and haematoxylin. Migration was quantified using 6 standardized microscopy fields per membrane.

### Invasion assay

MB231/miLacZ and MB231/miANGPTL2 cells were cultured in 96-well plates (ESSEN ImageLock plates) according to the manufacturer's instructions (ESSEN IncuCyte ZOOM) and visualized using a real-time cell imaging system (IncuCyte™ live-cell, ESSEN BioScience, Inc). In brief, plates were coated with a thin layer (50 μl) of collagen-1 (300 μg/ml) (R&D System) and placed in a 37°C incubator with 5% CO_2_ overnight. Then, 100 μl of the cell suspension (1 × 10^5^ cells/ml) was added to each well, plates were incubated 6 h, and a scratch was made in the plate using a 96-pin WoundMaker™. 50 μl of a 5× neutralization solution (DMEM with 1.875% bicarbonate buffer and 25% FBS) plus a working stock of 200 μl of 3.75 mg/ml collagen-I in 20 mM acetic acid were mixed and 100 μl of the mixture was added to each plate for 30 min. Then, 100 μl of culture media was added to each. Images were automatically acquired and registered by the IncuCyte™ software system (ESSEN BioScience). Typical kinetic updates were recorded at 2 h intervals over a 24 h period. CXCL12 (200 ng/ml final concentration, R&D Systems) was added to the neutralization solution with or without 20 μM of the MKK inhibitor U0126 (Merck KGaA, Darmstadt, Germany).

### Immunoblot analysis

Immunoblot analysis was performed as described^24^. SuperSep Ace gels (5–20%) were from Wako. Antibodies were against ETS1 (Abcam, Cambridge, UK), HSC70 (B-6, Santa Cruz Biotechnology), CXCR4 (ab124824, ab2074; Abcam), and ANGPTL2 (R&D Systems). Immunodetection was performed using an enhanced chemiluminescence (ECL) kit (GE Healthcare, Amersham, UK).

### CXCL12-stimulated ERK1/2 phosphorylation

Cells were serum-starved overnight and stimulated with 100 ng/ml CXCL12 (R&D Systems) for 5, 10 or 20 min at 37°C. Cells were lysed as in the immunoblot analysis. Rabbit polyclonal antibodies to phospho-ERK1/2 and ERK1/2 were used for immunodetection (Cell Signaling Technology, Danvers, MA, USA).

### Establishment of ANGPTL2-overexpressing cells

To create stably-transfected lines, MDA-MB231 or T47-D cells were transduced with FLAG-tagged ANGPTL2 expression^26^ or control vectors using Lipofectamine 2000 (Invitrogen), according to the manufacturer's protocol. Transfected lines were selected in 800 μg/ml G418 (Merck KGaA).

### *ETS1* knockdown

MB231/ANGPTL2 cells were reseeded in 6-well plates with two specific ETS1 siRNAs (SR301470A and SR301470B; ORIGENE, ETS1 (Locus ID 2113) Trilencer-27 Human siRNA) or control Trilencer-27 Universal Scrambled Negative Control (ORIGENE, Rockville, MD, USA). Total protein was extracted for immunoblot analysis, and ETS1 protein expression was analyzed after 48 h of incubation. FACS analysis was undertaken after 48 h of incubation.

### Establishment of luciferase-expressing cells

For bioluminescence imaging, *ANGPTL2*-knockdown MDA-MB231 cells were transfected with pGL4.51 (luc/CMV/Neo) vector^26^. Stable lines were selected in G418 (800 μg/ml). *ANGPTL2*-overexpressing MDA-MB231 cells were transfected with pGL4.50 (luc/CMV/Hygro) vector^15^. Stable lines were selected in hygromycin B (200 μg/ml)(Invitrogen).

### Quantitation of ANGPTL2 protein by ELISA

MB231/Control/luc, MB231/ANGPTL2/luc, MB231/miLacZ/luc, or MB231/miANGPTL2/luc cells were grown to confluency. The medium was then changed, cells were maintained for another 24 h, and medium was collected to quantify ANGPTL2 protein by ELISA using the ANGPTL2 Assay Kit (IBL, Fujioka, Gunma, Japan), according to the manufacturer's instructions.

### Animal studies

All experiments were performed according to guidelines of Institutional Animal Committee of Kumamoto University. For intracardiac injections, subconfluent tumor cells were harvested, washed in PBS, and resuspended at a concentration of 1 × 10^6^ cells/ml. Non obese diabetic/severe combined immunodeficient (NOD/SCID) Janus kinase 3 knockout (NOJ) mice^26^ were anesthetized by isoflurane and placed in the supine position. With a 29-gauge needle, 1 × 10^5^ cells were injected into the left ventricle of anesthetized mice after visualization of arterial blood flow into the syringe. Metastasis was monitored by bioluminescence imaging for 5 weeks after injection. D-Luciferin (100 μg/g) was injected into back skin of anesthetized mice before imaging, and images were then acquired using a NightOWL II LB 983 System (Berthold Technologies, Bad Wildbad, Germany). Luminescence was calculated using IndiGO2 software. For the orthotopic implantation model, subconfluent tumor cells were harvested, washed in PBS, and resuspended at 1 × 10^7^ cells/ml. Female 8–12 week-old NOJ mice were anesthetized by isoflurane, and for the breast cancer model, cells (1 × 10^6^) in 100 μl PBS were injected into their mammary fat pads.

### Human studies

The study group included 181 specimens of female primary invasive duct breast cancer cases from patients diagnosed between January of 1989 and December of 1996 at Kumamoto University. This study was approved by the Ethics Committees of Kumamoto University. Written informed consent was obtained from each subject.

### Immunohistochemical staining

Immunohistochemical analysis was performed as described^26^. Specimens were incubated with rabbit polyclonal anti-human ANGPTL2 (Abcam) or anti-CXCR4 (Abcam) antibodies. Estrogen receptor (ER), progesterone receptor (PgR) and HER2 receptor analysis was performed as described^53^.

### Evaluation of immunostaining

Each patient sample was evaluated by two independent investigators blinded to the clinical pathology parameters. We divided the 181 total samples (including 178 assessed for MMP-13, as 3 were excluded due to sample loss) into two groups, based on the median percentage of ANGPTL2-, CXCR4- or MMP-13-positive invasive cells. ANGPTL2- or MMP-13 positive groups were defined as having ≥50% positive cells; the CXCR4-positive group was defined as having ≥25% positive cells.

### Treatment of cells with integrin α5β1 antibody

Parental MDA-MB231 cells were maintained in serum-free Leibovitz's L15 for 24 hours. Then cells were incubated with α5β1 antibody (25 μg/ml) for 24 hours.

Total RNA from cells was extracted with TRIzol reagent (Invitrogen). Deoxyribonuclease-treated RNA was reverse-transcribed with a PrimerScript RT Reagent Kit (Takara Bio). PCR was performed using SYBR Premix Ex Taq II (Takara Bio).

### Statistics

Analyses were performed using GraphPad Prism (GraphPad Software, CA, USA). The Kaplan-Meier log-rank test was used to analyze mouse survival. Correlations between ANGPTL2, CXCR4 and MMP-13 were tested via Fisher's exact test. Distant relapse-free survival (DRFS) was estimated through the Kaplan-Meier method and compared between groups using the log-rank test.

## Author Contributions

T.M., E.M. and O.Y. developed the initial concept. T.M., E.M. and O.Y. designed experiments. T.M., O.H. and T.K. performed experiments and analyzed data. M.S.M. and I.M. performed RNA sequencing. Y.Y. analyzed clinical data. T.M., M.E. and O.Y. co-wrote the manuscript. T.N., H.T., H.I., M.Y., K.M., J.M., H.H., I.M. and K.T. reviewed the manuscript. H.I. and H.M. supervised the study. All authors discussed the results and commented on the manuscript.

## Supplementary Material

Supplementary Information Supplementary Info
