# Supplementary material for: ANGPTL2 increases bone metastasis of breast cancer cells through enhancing CXCR4 signaling
Source: Sci Rep. 2015 Mar 16;5:9170. doi: 10.1038/srep09170 (PMC4360633; doi:10.1038/srep09170)
Supplement: Supplementary Information — Supplementary Info [file srep09170-s1.pdf]

Supplementary information for

ANGPTL2 increases bone metastasis of breast cancer cells through enhancing CXCR4 signaling

Tetsuro Masuda, Motoyoshi Endo, Yutaka Yamamoto, Haruki Odagiri, Tsuyoshi Kadomatsu, Takayuki Nakamura, Hironori Tanoue, Hitoshi Ito, Masaki Yugami, Keishi Miyata, Jun Morinaga, Haruki Horiguchi, Ikuyo Motokawa, Kazutoyo Terada, Masaki Suimye Morioka, Ichiro Manabe, Hirotaka Iwase, Hiroshi Mizuta and Yuichi Oike

This file includes:

Supplementary Figures and Tables legends

Supplementary Figure S1-S18

Supplementary Table 1-3

Supplementary information

Supplementary Figure S1

List of the top 10 genes up-regulated in MB231 cells following *ANGPTL2* knockdown, based on RNA sequencing analysis. Data are mean $\log_2$ value of fold-change relative to control MB231/miLacZ cells.

Supplementary Figure S2

(A) Full length western blots of data presented in Figure 1B. Lane 1 represents an MB231/miLacZ cell lysate and Lane 2 represents an MB231/miANGPTL2 cell lysate. (B) Representative image of western blot analysis of MB231/miLacZ and MB231/miANGPTL2 cells using a CXCR4 antibody (ab2074) different from the one used in A. (C) Relative *CXCL12* expression in MB231/ANGPTL2 cells. Data are means ±SEM from three experiments.

Supplementary Figure S3

(A-C) Transwell migration assay of MB231/miLacZ and MB231/miANGPTL2 cells in the presence of several CXCL12 concentrations. (D) Representative image of a transwell migration assay of MB231/miANGPTL2 and MB231/miLacZ cells 18 h after CXCL12 treatment. Migrated cells were fixed with 4% paraformaldehyde and stained for 30 min with Giemsa stain (Wako). Scale bar = 100 μm.

Supplementary Figure S4

Time course of invasion activity of MB231/miANGPTL2 and MB231/miLacZ

cells with or without CXCL12 treatment. Data are means ±SEM from five experiments; ⋆P<0.05 (unpaired two-tailed Student's *t*-test).

Supplementary Figure S5

Full length western blots representing data shown in Figure 2E. Lane 1 represents an MB231/miLacZ cell lysate and Lane 2 represents an MB231/miANGPTL2 lysate.

Supplementary Figure S6

Time course showing representative immunoblotting of indicated cells for phosphorylated ERK1/2 (p-ERK), total ERK1/2 and HSC70 after CXCL12 treatment.

Supplementary Figure S7

(A) Full length western blots of data shown in Figure 3A. Lane 1 represents an MB231/miLacZ cell lysate and Lane 2 represents an MB231/miANGPTL2 lysate. (B) Representative image showing immunoblot of MB231/Control and MB231/ANGPTL2 cells for ANGPTL2 by anti-ANGPTL2 antibody. Lane 1 represents an MB231/Control cell lysate and Lane 2 represents an MB231/ANGPTL2 lysate. Open arrowhead indicates FLAG-tagged ANGPTL2; filled arrowhead indicates endogenous ANGPTL2. (C) Relative *CXCL12* expression in MB231/ANGPTL2 cells. Data are means ±SEM from three experiments. (D) Full length western blots of data shown in Figure 3C. Lane 1 represents an MB231/Control cell lysate and Lane 2 represents an MB231/ANGPTL2 lysate.

Supplementary Figure S8

Full length western blots of data shown in in Figure 3G. Lane 1 represents an MB231/ANGPTL2 ETS1 siRNA-Control cell lysate, Lane 2 an MB231/ANGPTL2 ETS1 siRNA-1 lysate, and Lane 3 an MB231/ANGPTL2 ETS1 siRNA-2 lysate.

Supplementary Figure S9

(A) Relative ANGPTL2 expression in T47-D/ANGPTL2 cells. Data are means ±SEM from three experiments. ★★*P*<0.01(unpaired two-tailed Student's *t*-test). (B) Relative CXCR4 expression in T47-D/ANGPTL2 cells. Data are means ±SEM from three experiments. ★★*P*<0.01(unpaired two-tailed Student's *t*-test). (C) Relative ETS1 expression in T47-D/ANGPTL2 cells. Data are means ±SEM from three experiments; ★★*P*<0.01(unpaired two-tailed Student's *t*-test).

(D) CXCR4 cell surface expression in T47-D/ANGPTL2 and T47-D/Control cells based on flow cytometry. Gray shaded area represents isotype control antibody group. T47-D/ANGPTL2 cells, dotted-line; T47-D/Control cells, solid line.

Supplementary Figure S10

Full length western blots of data shown in Figure 4A. Lane 1 represents an MB231/miLacZ/luc cell lysate and Lane 2 represents an MB231/miANGPTL2/luc lysate.

Supplementary Figure S11

(A) Relative number of proliferating MB231/miLacZ/luc and

MB231/miANGPTL2/luc cells after 24, 48 or 72 hours of culture in normoxic or hypoxic conditions. Data are relative to the number of cells present at seeding and are means ±SEM from five experiments. (B) Growth of mouse primary tumors derived from MB231/miLacZ/luc and MB231/miANGPTL2/luc cells, 3 weeks after implantation (n=6). Scale bar=10 mm.

Supplementary Figure S12

Additional images showing bioluminescence signals in xenografted mice and microscopy images of tumor cells shown in Figure 4. Left panels; bioluminescence signals were captured at indicated times after xenografting. Center and right panels, microscopy images of H&E-stained tumor cells metastasized to tibial bone located within the red circle seen in left figures, 4 weeks after injection of MB231/miLacZ/luc (top 3) or MB231/miANGPTL2/luc (bottom 3) cells. Right panels are magnifications of squares in center panels. Center image scale bar=1.0 mm; right image scale bar=100 μm.

Supplementary Figure S13

(A) Comparison of ANGPTL2 levels in the culture medium of indicated cells. Data are means ±SEM from three experiments; ⋆⋆P<0.01(unpaired two-tailed Student's *t*-test). (B) Representative images of bioluminescence signals in xenografted mice. MB231/Control/luc or MB231/ANGPTL2/luc cells were injected into the left cardiac ventricle of immunodeficient mice (n=5). At indicated times after xenografting, bioluminescence signals were captured. Images of weeks 1-4 are displayed on the same scale. (C) Kaplan-Meier survival curves of mice bearing tumors derived from MB231/Control/luc (n=8) or

MB231/ANGPTL2/luc (n=8) cells. ⋆*P*<0.05 (log-rank test).

Supplementary Figure S14

(A) Relative *CXCR4* expression in MB231/ANGPTL2/miLacZ/luc and MB231/ANGPTL2/miCXCR4/luc cells. Data are means ±SEM from three experiments; ⋆*P*<0.05(unpaired two-tailed Student's *t*-test). (B) CXCR4 cell surface expression in MB231/ANGPTL2/miLacZ/luc and MB231/ANGPTL2/miCXCR4/luc cells based on flow cytometry. Gray shaded area represents isotype control antibody group. MB231/ANGPTL2/miCXCR4/luc cells, dotted-line; MB231/ANGPTL2/miLacZ/luc cells, solid line. (C) MB231/ANGPTL2/miLacZ/luc and MB231/ANGPTL2/miCXCR4/luc cells were injected into the left cardiac ventricle of immunodeficient mice (n=6). At indicated times after xenografting, bioluminescence signals were captured. Images are displayed on the same scale. (D) Representative microscopy images of H&E-stained tumor cells metastasized to tibial bone, 3 weeks after injection with MB231/ANGPTL2/miLacZ/luc (left 3) or MB231/ANGPTL2/miCXCR4/luc (right 3) cells. Black arrowhead indicates tumor cells. Lower panels are magnifications of squares in upper panels. Upper image scale bar=1.0 mm; lower image scale bar=100 µm.

Supplementary Figure S15

(A) ANGPTL2 and MMP-13 immunostaining within primary tumors derived from patients. Representative images of ANGPTL2-negative and MMP-13-negative (Pt. 3) and ANGPTL2-positive and MMP-13-positive (Pt.4) specimens. Scale bar=100 μm. (B) Distribution of ANGPTL2 and MMP-13 staining in tumor

specimens from breast cancer patients. ★★*P*<0.001 (Fisher's exact test).

Supplementary Figure S16

(A) CXCR4 and MMP-13 immunostaining within patient primary tumors. Representative images of CXCR4-negative and MMP-13-negative (Pt.5) and CXCR4-positive and MMP-13-positive (Pt.6) specimens. Scale bar=100 μm. (B) Distribution of ANGPTL2 and MMP-13 staining in tumor specimens from breast cancer patients. ★★*P*<0.001 (Fisher's exact test).

Supplementary Figure S17

(A) Cohort of probability of distant relapse-free survival in ANGPTL2-positive (n=88) and ANGPTL2-negative (n=93) groups. (B) Cohort of probability of distant relapse-free survival in CXCR4-positive (n=75) and CXCR4-negative (n=106) groups. (C) Cohort of probability of distant relapse-free survival in MMP-13-positive (n=89) and MMP-13-negative (n=89) groups.

Supplementary Figure S18

(A) Cell surface integrin α5β1 expression in MB231/Control and MB231/ANGPTL2 cells based on flow cytometry. Gray shaded area represents isotype control antibody group. MB231/Control cells, dotted-line; MB231/ANGPTL2 cells, solid line. (B) LILRB2 cell surface expression in MB231/Control and MB231/ANGPTL2 cells based on flow cytometry. Gray shaded area represents isotype control antibody group. MB231/Control cells, dotted-line; MB231/ANGPTL2 cells, solid line. (C) Relative *CXCR4* expression in MB231/Control and MB231/ANGPTL2 cells, 24h after treatment with or

without an anti-α5β1 antibody. Data from MB231/ANGPTL2 cells was set at 1. Data are means ±SEM from five experiments; ★$P<0.05$, ★★$P<0.01$(unpaired two-tailed Student's *t*-test).

Supplementary Table 1.

Sequences of primers used for quantitative RT-PCR.

Supplementary Table 2.

Correlation of primary tumor ANGPTL2 expression and patient characteristics. ER indicates estrogen receptor. PgR indicates progesterone receptor. ★★ $P<0.001$(Fisher's exact test). ††$P<0.001$(Pearson's chi-square test).

Supplementary Table 3.

Correlation of primary tumor CXCR4 expression and patient characteristics. ER indicates estrogen receptor. PgR indicates progesterone receptor. ★★ $P<0.001$(Fisher's exact test).

Symbol	Mean $\log_2$ (fold change) of transcripts
ZNF542	7.541
LOC339535	6.828
ZNF662	6.286
COBL	6.076
SNAP25	6.057
CACNA2D4	5.765
INHBA	5.74
BEX2	4.972
CCL3	4.098
GPR176	4.078

Figure S1 (Masuda et al)

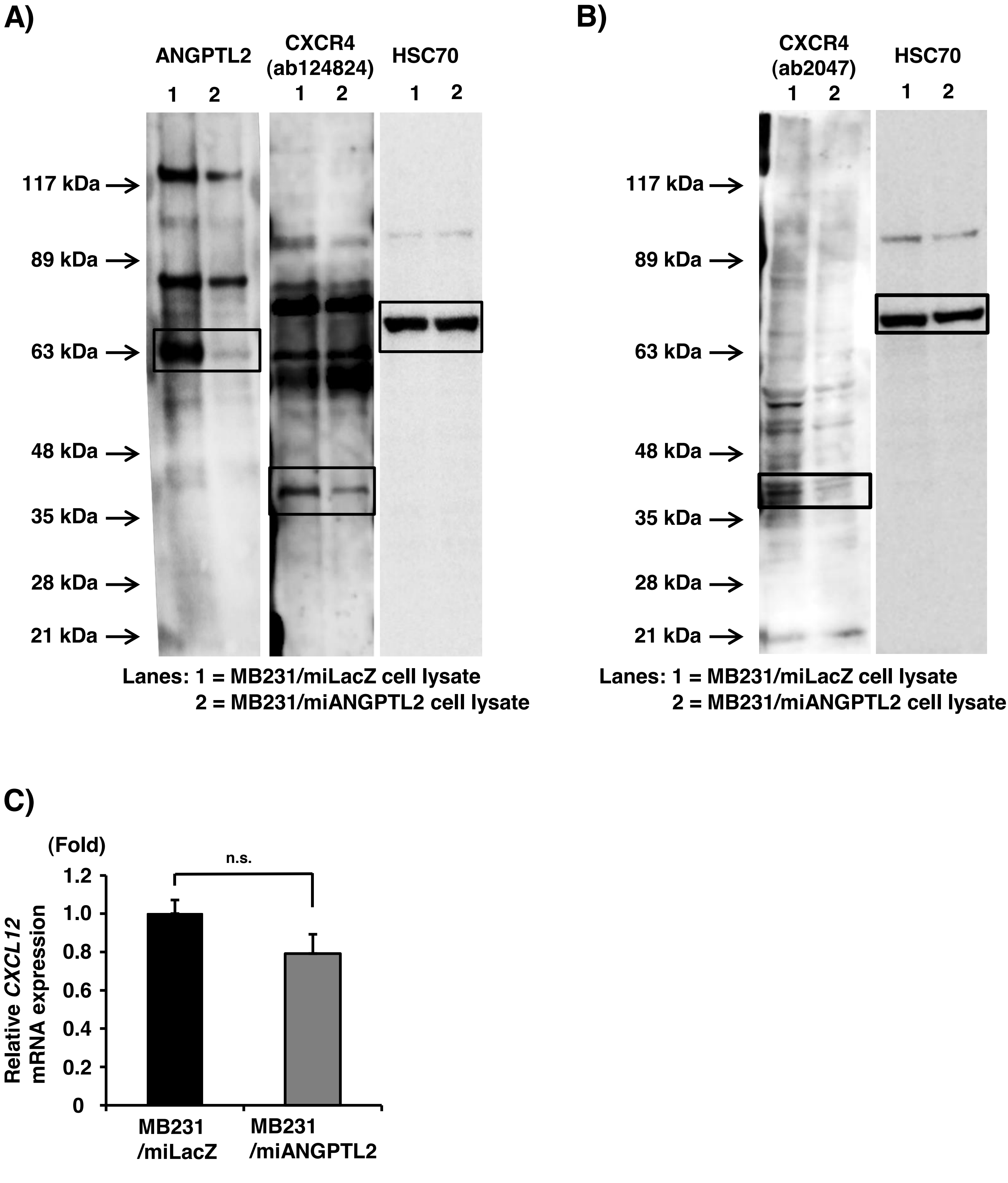

Figure S2 (Masuda et al)

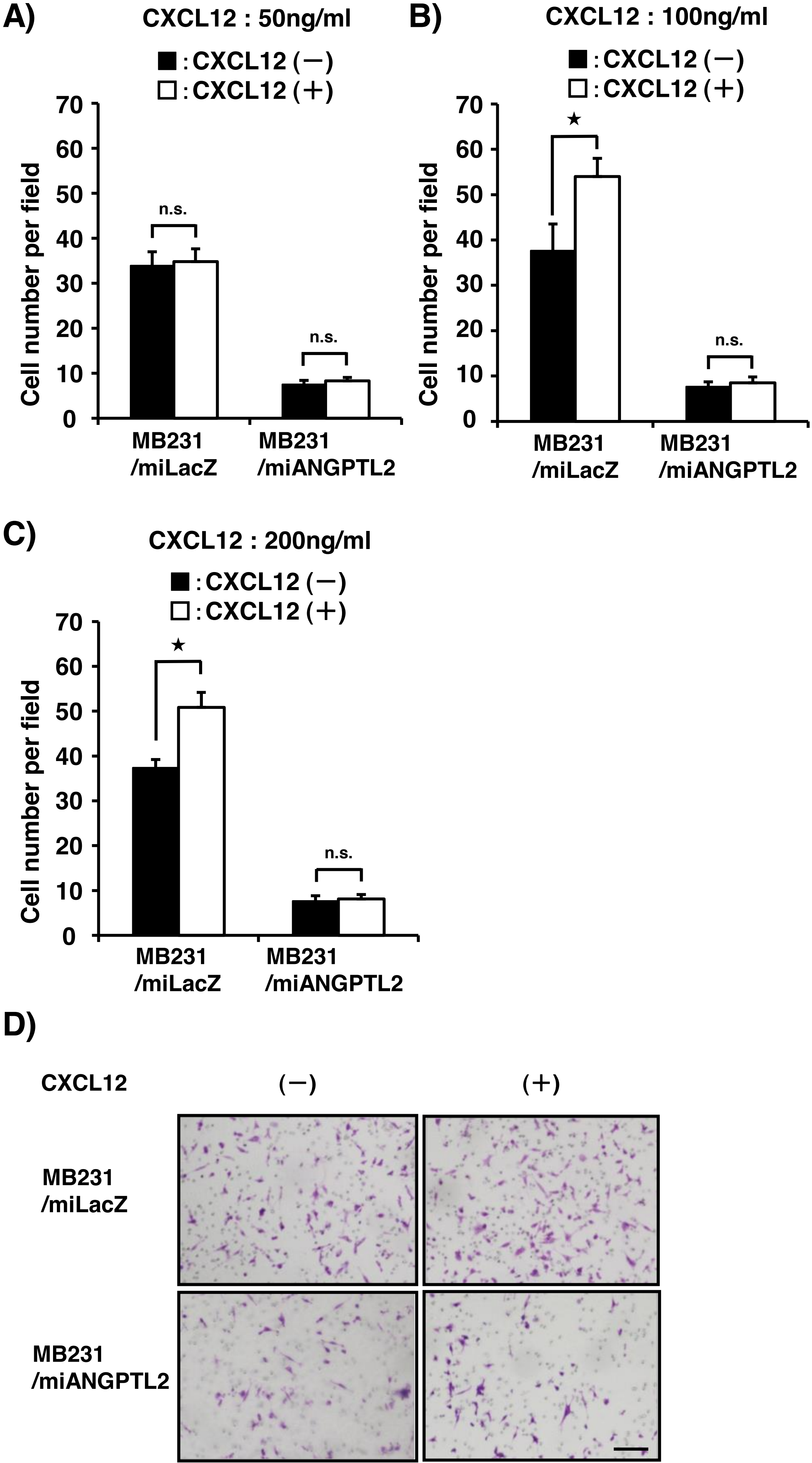

Figure S3 (Masuda et al)

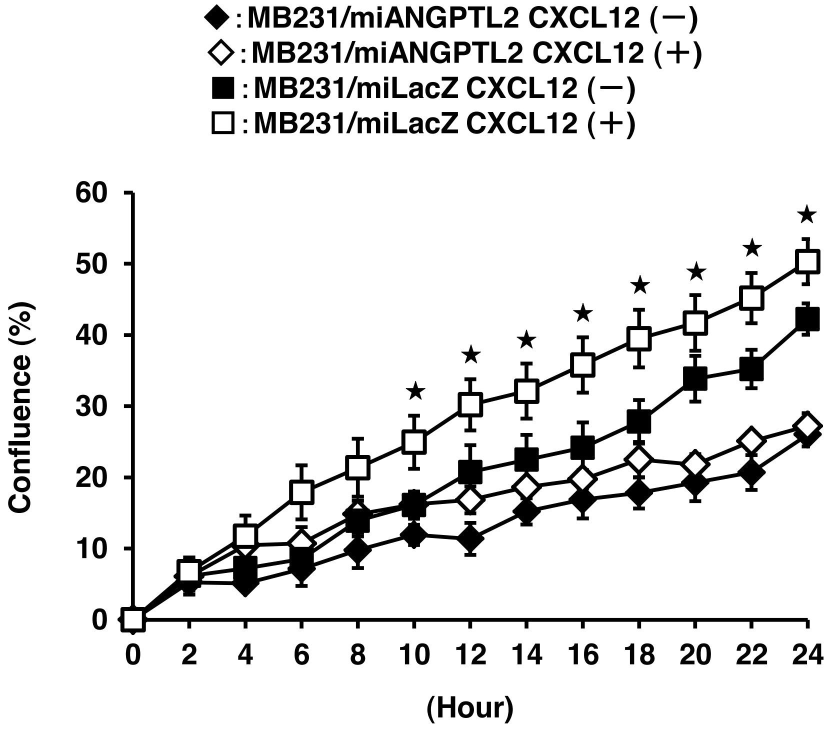

Figure S4 (Masuda et al)

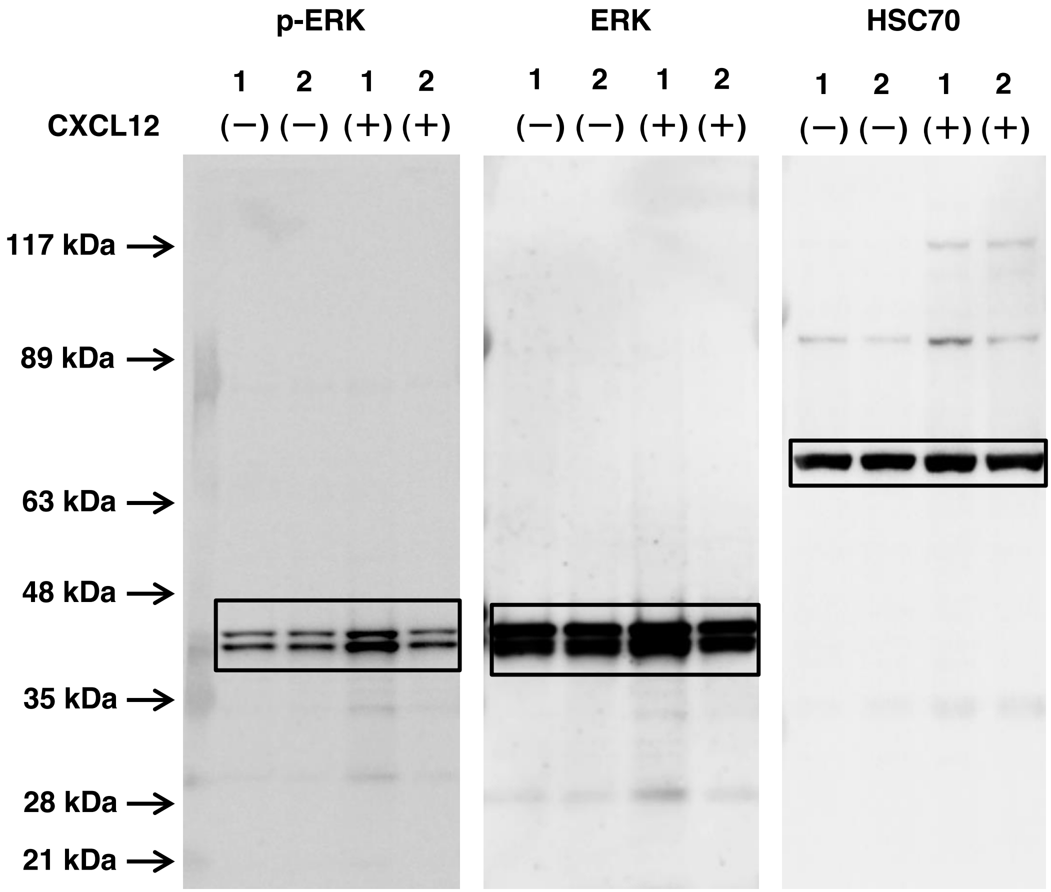

Lanes: 1 = MB231/miLacZ cell lysate
2 = MB231/miANGPTL2 cell lysate

Figure S5 (Masuda et al)

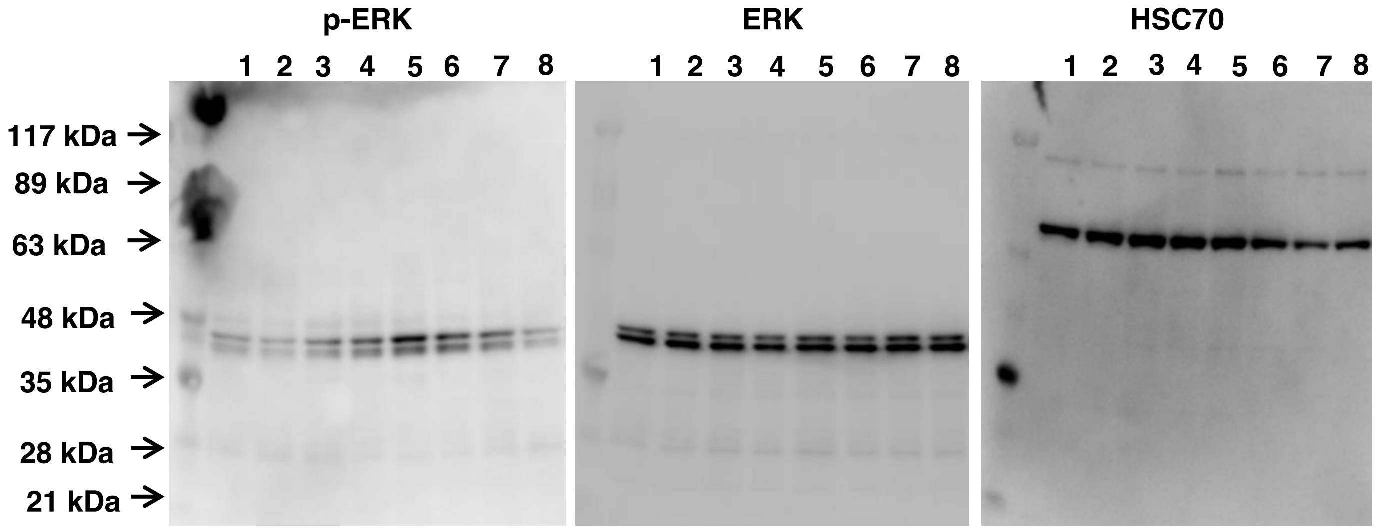

Lanes: 1 = MB231/miLacZ cell CXCL12 (−)
2 = MB231/miANGPTL2 cell CXCL12 (−)
3 = MB231/miLacZ cell CXCL12 (+) after 5min.
4 = MB231/miANGPTL2 cell CXCL12 (+) after 5min.
5 = MB231/miLacZ cell CXCL12 (+) after 10min.
6 = MB231/miANGPTL2 cell CXCL12 (+) after 10min.
7 = MB231/miLacZ cell CXCL12 (+) after 20min.
8 = MB231/miANGPTL2 cell CXCL12 (+) after 20min.

Figure S6 (Masuda et al)

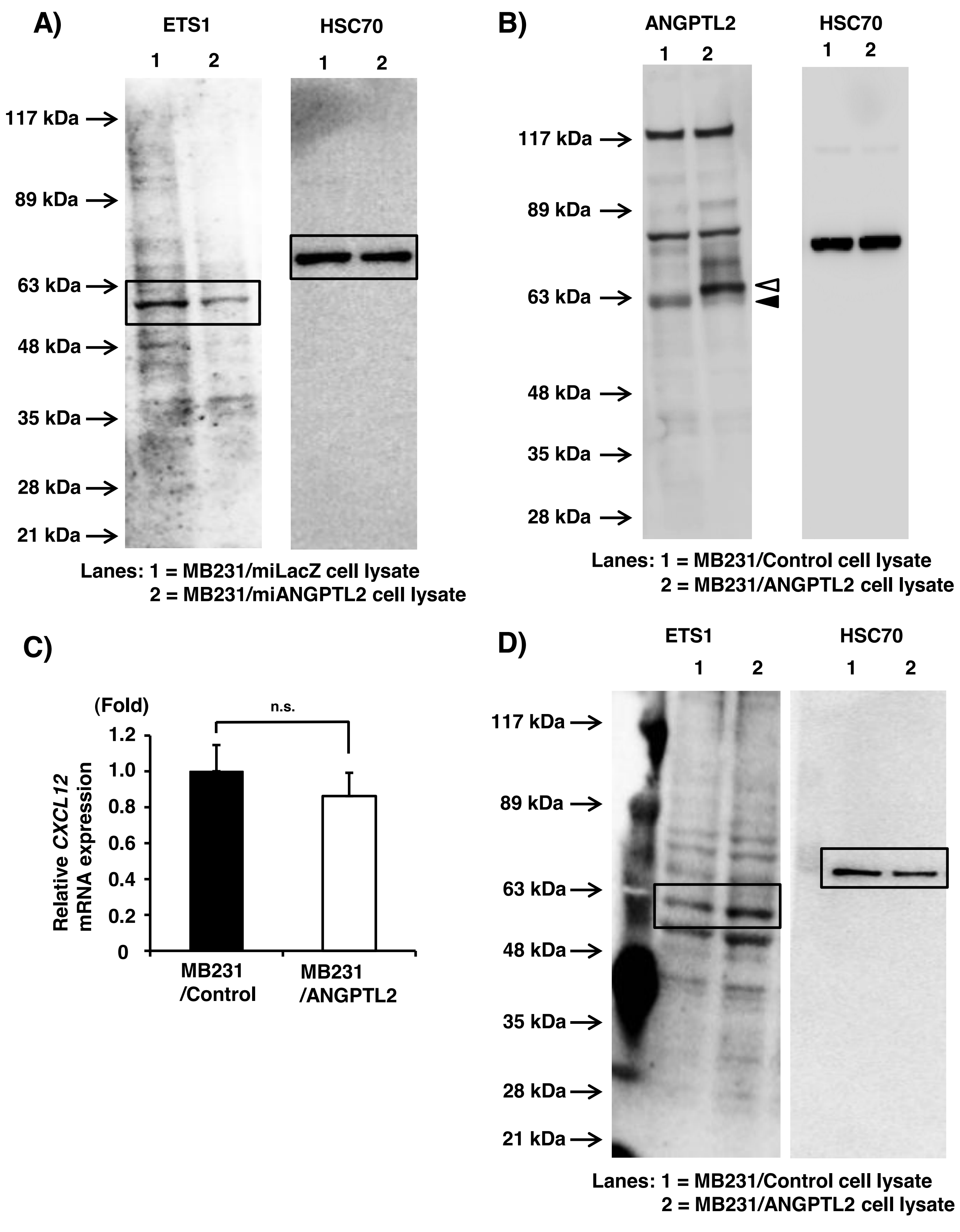

Figure S7 (Masuda et al)

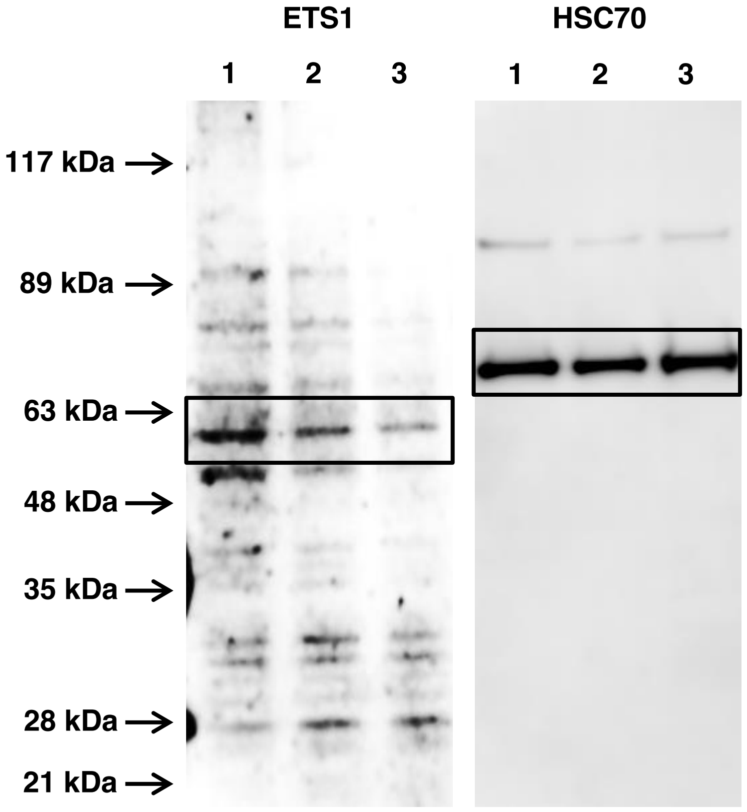

Lanes: 1 = MB231/ANGPTL2 ETS1 siRNA-Control cell lysate
2 = MB231/ANGPTL2 ETS1 siRNA-1 cell lysate
3 = MB231/ANGPTL2 ETS1 siRNA-2 cell lysate

Figure S8 (Masuda et al)

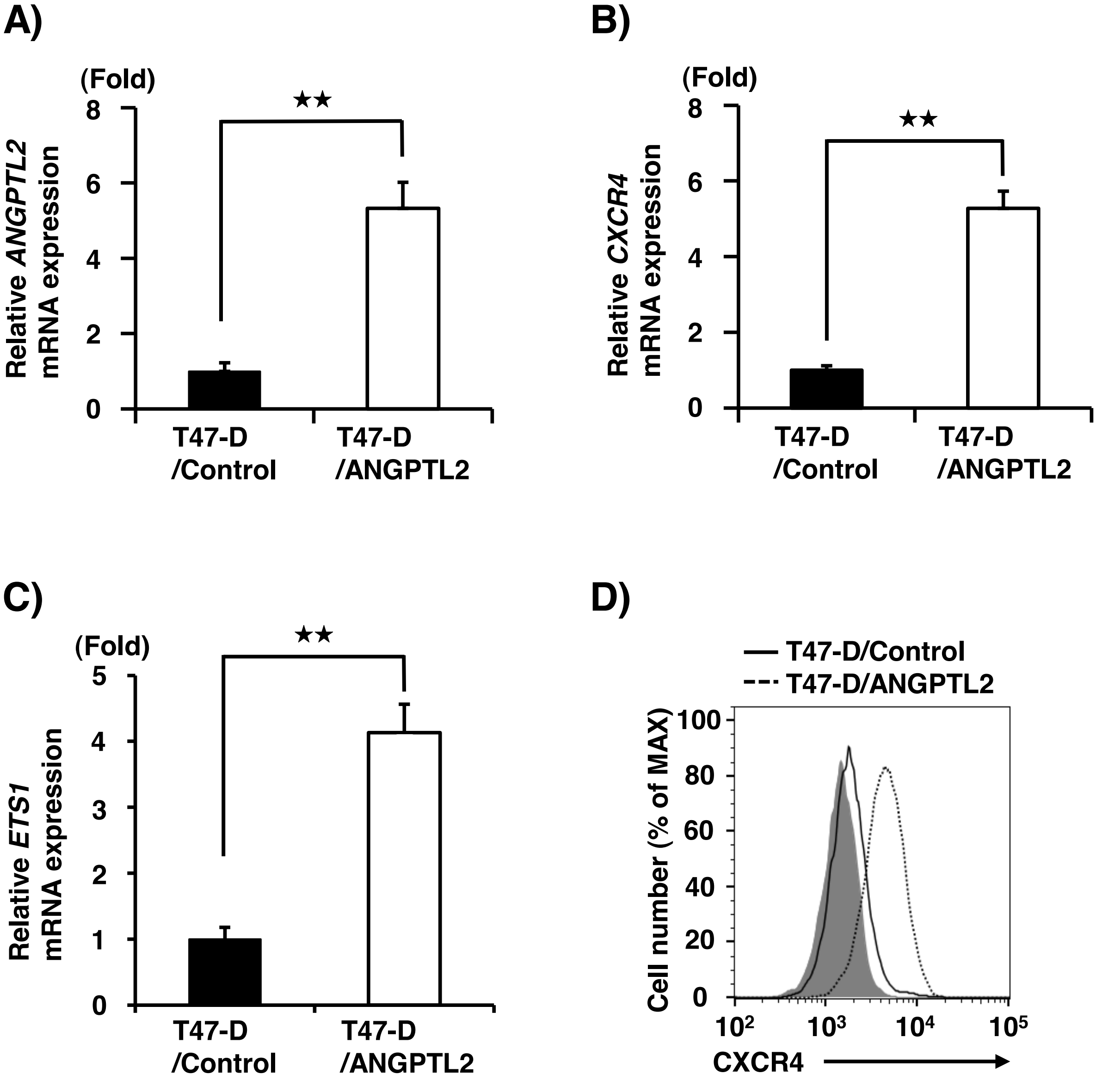

Figure S9 (Masuda et al)

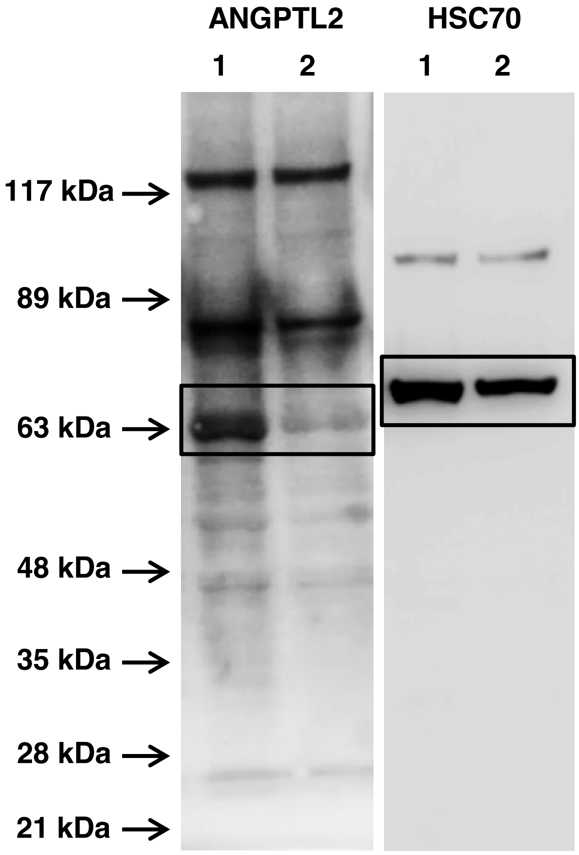

Lanes: 1 = MB231/miLacZ/luc cell lysate
2 = MB231/miANGPTL2/luc cell lysate

Figure S10 (Masuda et al)

A)

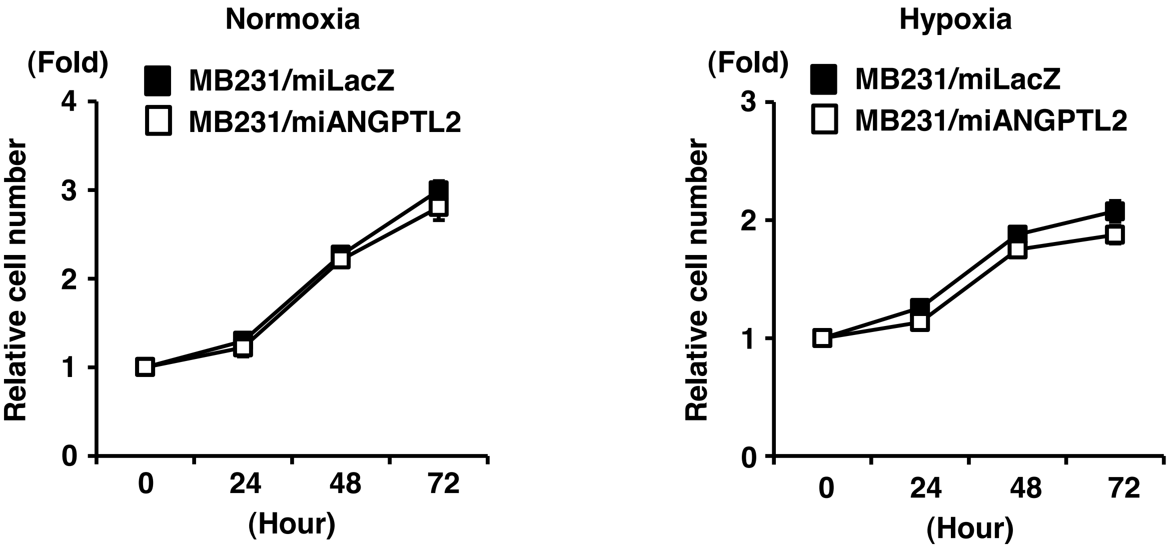

B)

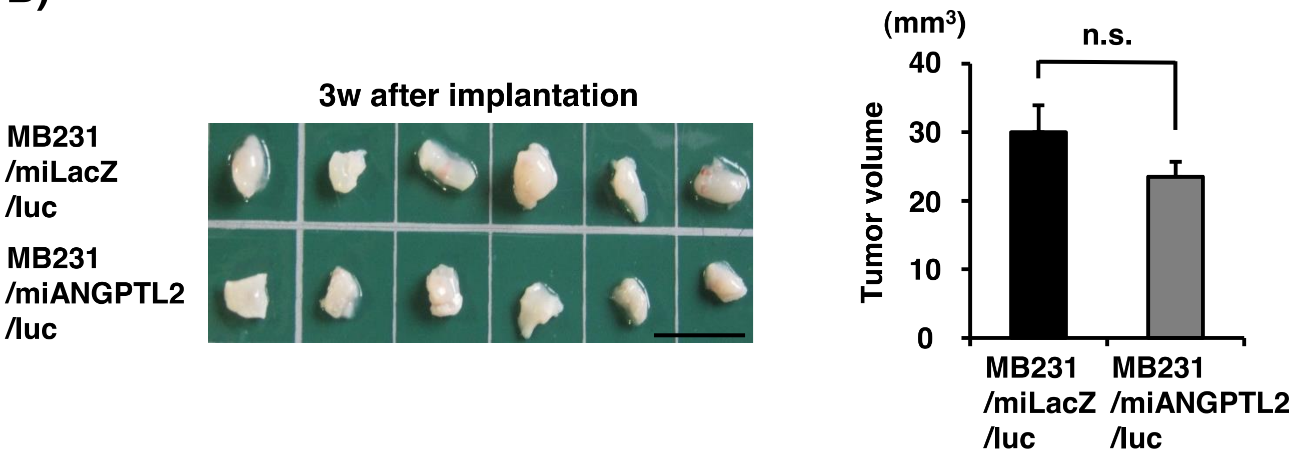

Figure S11 (Masuda et al)

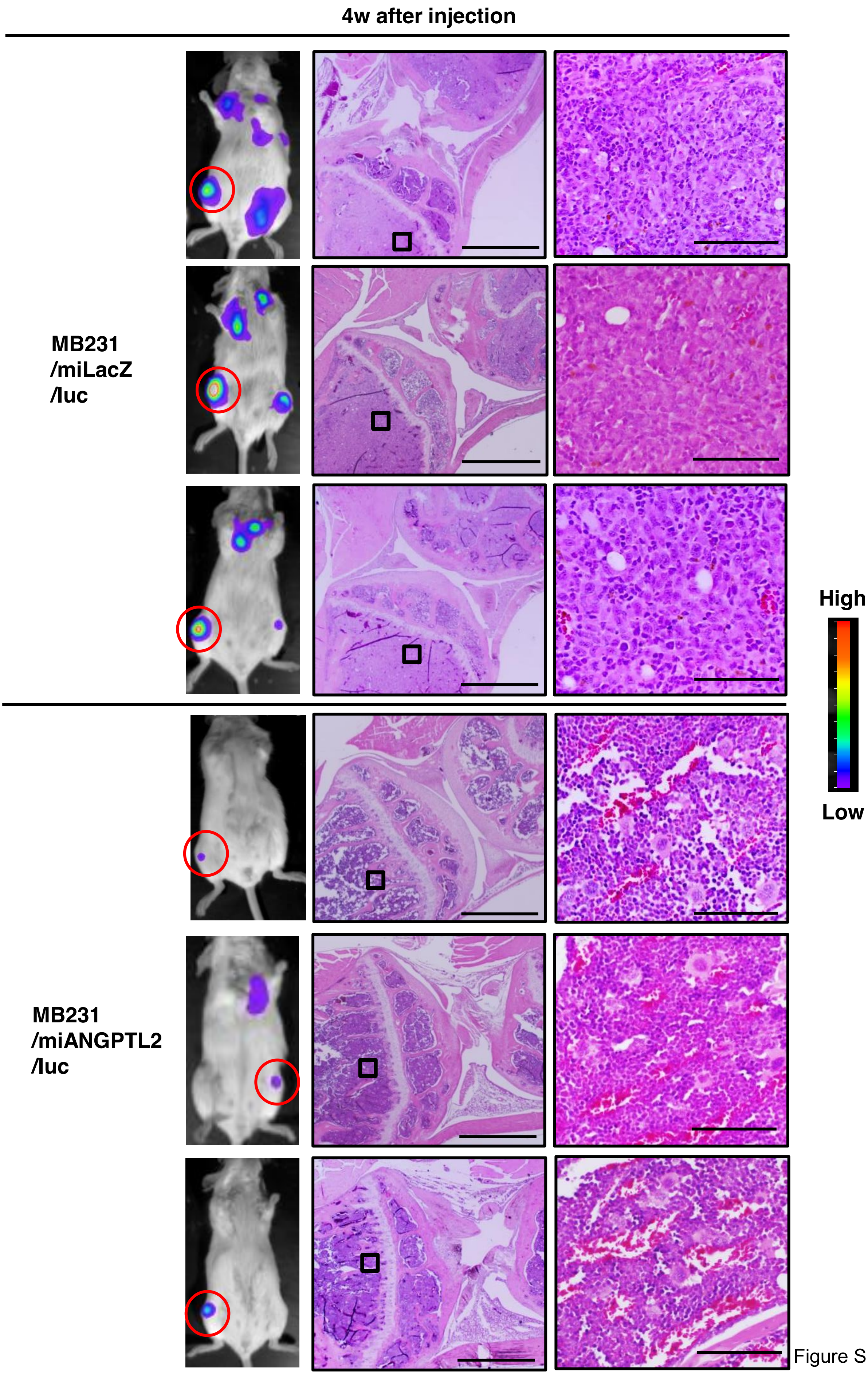

Figure S12 (Masuda et al)

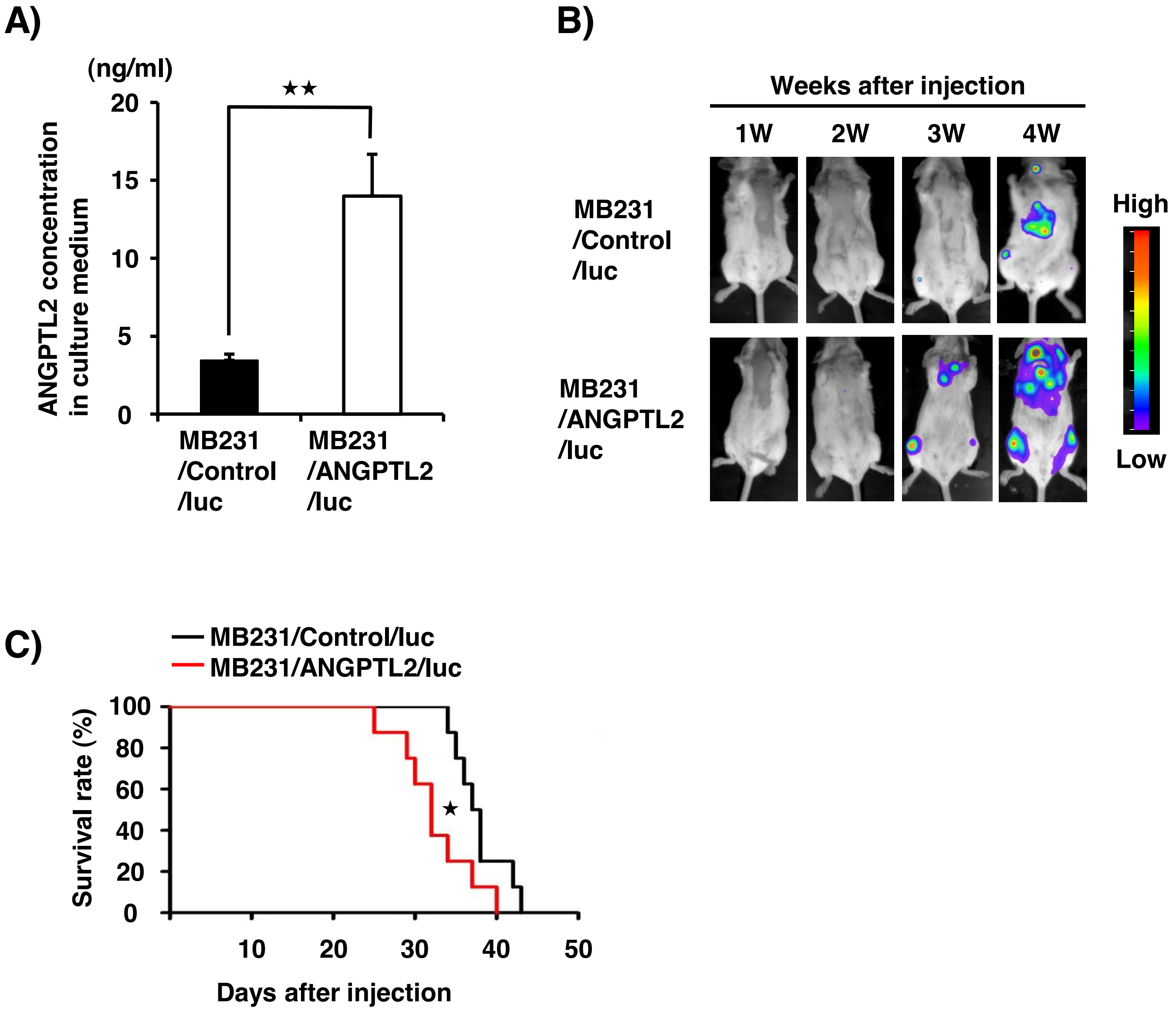

Figure S13 (Masuda et al)

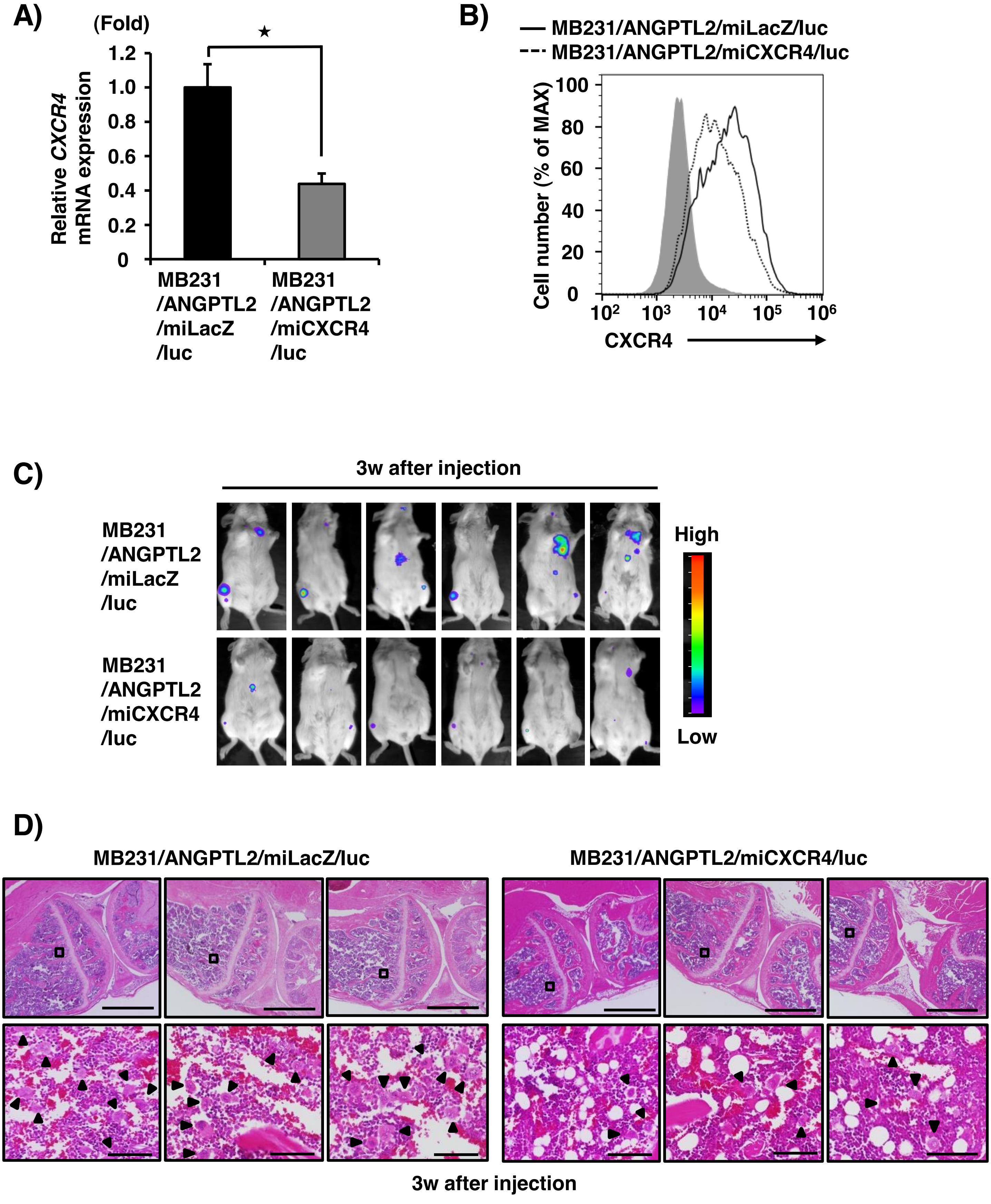

Figure S14 (Masuda et al)

A)

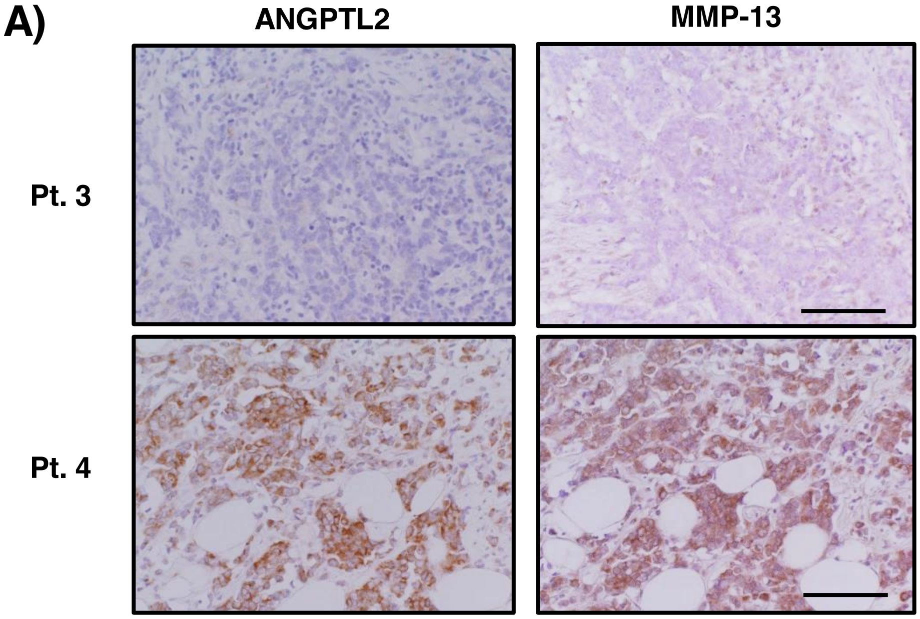

B)

	ANGPTL2 expression		
	Negative :n (%)	Positive :n (%)	*P*
MMP-13			<0.001★★
Negative (n=89)	60 (67.4)	29 (32.6)	
Positive (n=89)	31 (34.8)	58 (65.2)	

A)

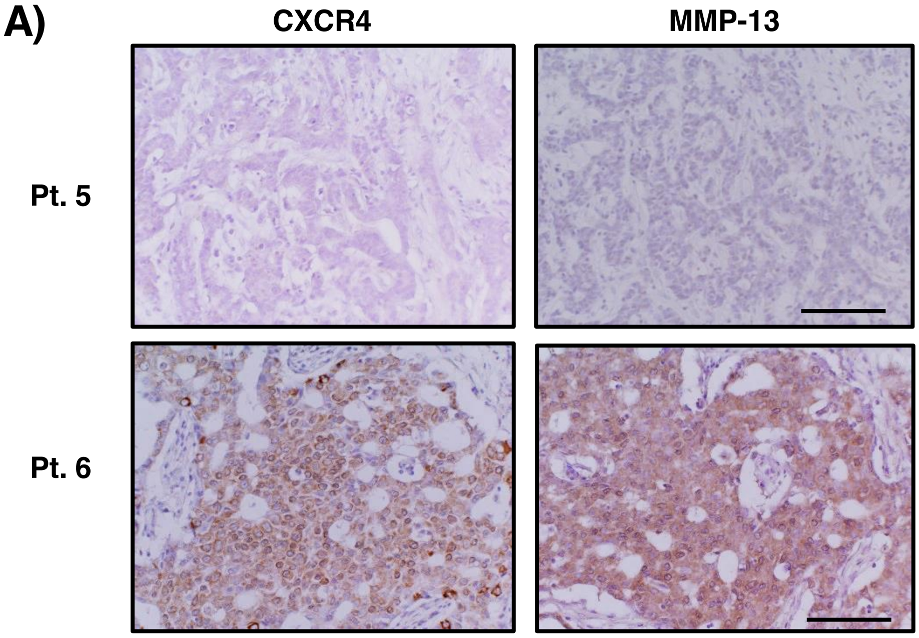

B)

	CXCR4 expression		
	Negative :n (%)	Positive :n (%)	*P*
MMP-13			<0.001★★
Negative (n=89)	67 (75.3)	22 (24.7)	
Positive (n=89)	37 (41.6)	52 (58.4)	

Figure S16 (Masuda et al)

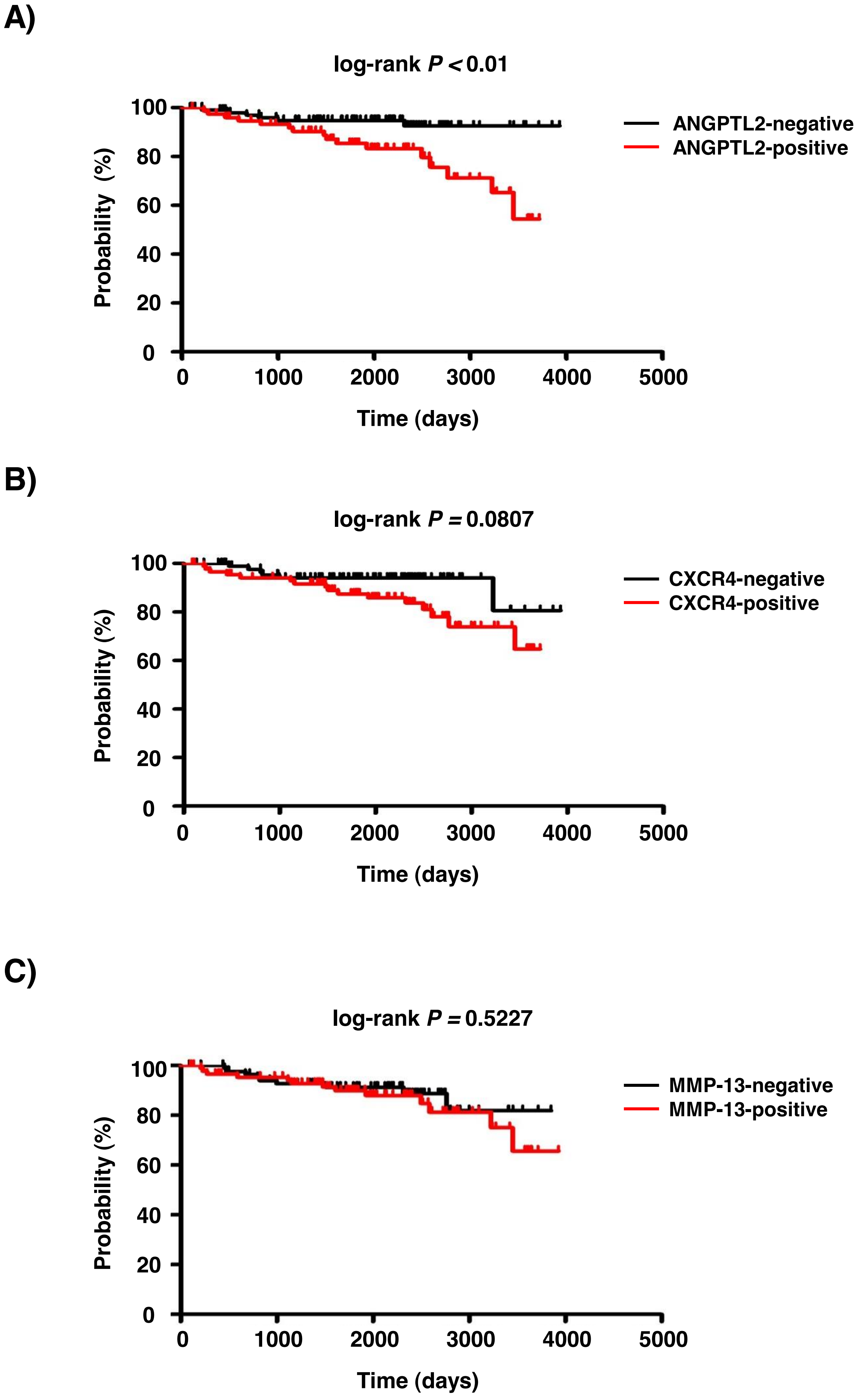

Figure S17 (Masuda et al)

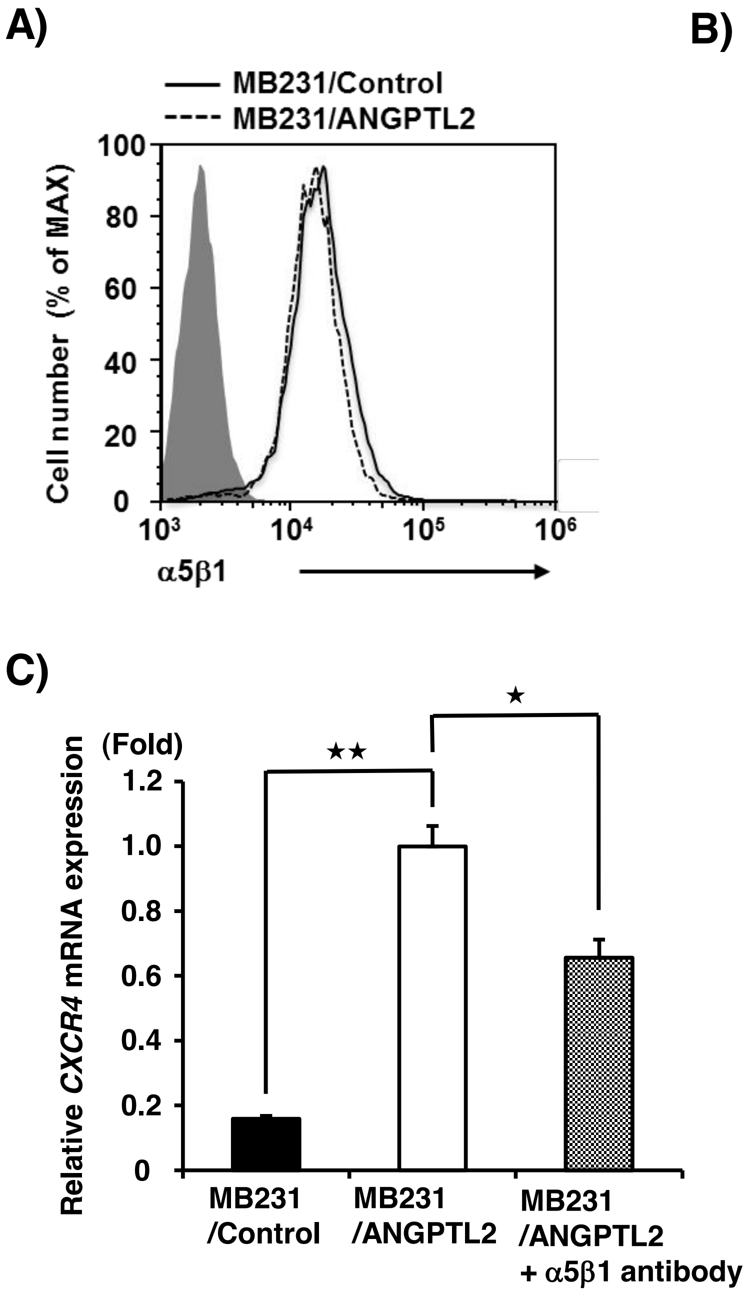
A)
MB231/Control
MB231/ANGPTL2
Cell number (% of MAX)
100
80
60
40
20
0
10³
10⁴
10⁵
10⁶
α5β1
B)
MB231/Control
MB231/ANGPTL2
Cell number (% of MAX)
100
80
60
40
20
0
10³
10⁴
10⁵
10⁶
LILRB2
C)
Relative CXCR4 mRNA expression
(Fold)
1.2
1.0
0.8
0.6
0.4
0.2
0
★★
★
MB231
/Control
MB231
/ANGPTL2
MB231
/ANGPTL2
+ α5β1 antibody

Supplementary Table 1.

Sequences of primers used in quantitative RT-PCR

Gene		Sequences
ANGPTL2	Forward	AGTTTCCGCCTGGAACCTGAG
	Reverse	GGAGTGGGCACAGGCGTTATAC
CXCR4	Forward	CTCCAGTAGCCACCGCATCA
	Reverse	TCCTCGGTGTAGTTATCTGAAGT
CXCL12	Forward	AAGCCCGTCAGCCTGAGCTA
	Reverse	TTAGCTTCGGGTCAATGCACAC
MMP-13	Forward	TTGATGATGATGAAACCTGGACAAG
	Reverse	TTGCCGGTGTAGGTGTAGATAGGAA
RSP18	Forward	TTTGCGAGTACTCAACACCAACATC
	Reverse	GAGCATATCTTCGGCCCACAC

Supplementary Table 2.

	ANGPTL2 expression		
	Negative :n (%)	Positive :n (%)	*P*
Age			0.8480
＜50	17 (53.1)	15 (46.9)	
≧50	76 (51.0)	73 (49.0)	
T (Primary tumor)			＜0.001††
T1	69 (65.7)	36 (34.3)	
T2	22 (34.4)	42 (65.6)	
T3＋4	2 (16.7)	10 (83.3)	
Nuclear grade			0.0731
Ⅰ	57 (59.4)	39 (40.6)	
Ⅱ	20 (42.6)	27 (57.4)	
Ⅲ	16 (42.1)	22 (57.9)	
Lymph node metastasis			＜0.001★★
Negative	81 (64.3)	45 (35.7)	
Positive	12 (21.8)	43 (78.2)	
Stage			＜0.001††
Ⅰ	61 (70.9)	25 (29.1)	
Ⅱ	30 (38.5)	48 (61.5)	
Ⅲ	2 (11.8)	15 (88.2)	
ER			0.2920
Negative	18 (43.9)	23 (56.1)	
Positive	75 (53.6)	65 (46.4)	
PgR			0.3614
Negative	38 (55.9)	30 (44.1)	
Positive	55 (48.7)	58 (51.3)	
HER2			0.1436
Negative	83 (53.9)	71 (46.1)	
Positive	10 (37.0)	17 (63.0)	

Supplementary Table 3.

	CXCR4 expression		
	Negative :n (%)	Positive :n (%)	*P*
**Age**			0.3246
＜50	16 (50.0)	16 (50.0)	
≧50	90 (60.4)	59 (39.6)	
**T (Primary tumor)**			0.1027
T1	77 (73.3)	28 (26.7)	
T2	39 (60.9)	25 (39.1)	
T3＋4	6 (50.0)	6 (50.0)	
**Nuclear grade**			0.3262
Ⅰ	61 (63.5)	35 (36.5)	
Ⅱ	24 (51.1)	23 (48.9)	
Ⅲ	21 (55.3)	17 (44.7)	
**Lymph node metastasis**			＜0.01★★
Negative	87 (69.0)	39 (31.0)	
Positive	19 (34.5)	36 (75.5)	
**Stage**			0.1538
Ⅰ	57 (66.3)	29 (33.7)	
Ⅱ	42 (53.8)	36 (46.2)	
Ⅲ	8 (47.1)	9 (52.9)	
**ER**			0.4770
Negative	22 (53.7)	19 (46.3)	
Positive	84 (60.0)	56 (40.0)	
**PgR**			0.4367
Negative	37 (54.4)	31 (45.6)	
Positive	69 (61.1)	44 (38.9)	
**HER2**			0.1381
Negative	94 (61.0)	60 (39.0)	
Positive	12 (44.4)	15 (55.6)	
